# Dosage-Sensitive Function of *RETINOBLASTOMA RELATED* and Convergent Epigenetic Control Are Required during the *Arabidopsis* Life Cycle

**DOI:** 10.1371/journal.pgen.1000988

**Published:** 2010-06-17

**Authors:** Amal J. Johnston, Olga Kirioukhova, Philippa J. Barrell, Twan Rutten, James M. Moore, Ramamurthy Baskar, Ueli Grossniklaus, Wilhelm Gruissem

**Affiliations:** 1 Department of Biology and Zurich-Basel Plant Science Center, ETH Zurich, Zurich, Switzerland; 2 Leibniz Institute of Plant Genetics and Crop Plant Research (IPK), Gatersleben, Germany; 3 Institute of Plant Biology and Zurich-Basel Plant Science Center, University of Zurich, Zurich, Switzerland; National Institute of Genetics, Japan

## Abstract

The plant life cycle alternates between two distinct multi-cellular generations, the reduced gametophytes and the dominant sporophyte. Little is known about how generation-specific cell fate, differentiation, and development are controlled by the core regulators of the cell cycle. In *Arabidopsis*, *RETINOBLASTOMA RELATED* (*RBR*), an evolutionarily ancient cell cycle regulator, controls cell proliferation, differentiation, and regulation of a subset of Polycomb Repressive Complex 2 (PRC2) genes and *METHYLTRANSFERASE 1* (*MET1*) in the male and female gametophytes, as well as cell fate establishment in the male gametophyte. Here we demonstrate that *RBR* is also essential for cell fate determination in the female gametophyte, as revealed by loss of cell-specific marker expression in all the gametophytic cells that lack *RBR*. Maintenance of genome integrity also requires *RBR*, because diploid plants heterozygous for *rbr* (*rbr/RBR*) produce an abnormal portion of triploid offspring, likely due to gametic genome duplication. While the sporophyte of the diploid mutant plants phenocopied wild type due to the haplosufficiency of *RBR*, genetic analysis of tetraploid plants triplex for *rbr* (*rbr/rbr/rbr/RBR*) revealed that *RBR* has a dosage-dependent pleiotropic effect on sporophytic development, trichome differentiation, and regulation of PRC2 subunit genes *CURLY LEAF* (*CLF*) and *VERNALIZATION 2* (*VRN2*), and *MET1* in leaves. There were, however, no obvious cell cycle and cell proliferation defects in these plant tissues, suggesting that a single functional *RBR* copy in tetraploids is capable of maintaining normal cell division but is not sufficient for distinct differentiation and developmental processes. Conversely, in leaves of mutants in sporophytic PRC2 subunits, trichome differentiation was also affected and expression of *RBR* and *MET1* was reduced, providing evidence for a *RBR*-PRC2-*MET1* regulatory feedback loop involved in sporophyte development. Together, dosage-sensitive *RBR* function and its genetic interaction with PRC2 genes and *MET1* must have been recruited during plant evolution to control distinct generation-specific cell fate, differentiation, and development.

## Author Summary

Understanding the convergent developmental mechanisms of core cell cycle genes is highly instructive in biology. When these genes are essential in development, lethality precludes mutation analysis throughout the life cycle of an organism. We subjected a homozygous lethal mutation in *RETINOBLASTOMA RELATED* (*RBR*) of *Arabidopsis* for tetraploid genetic analysis to study the function of *RBR* during the plant life cycle. In diploids, while *RBR–*deficient female gametophytes with features of aberrant cell fate and differentiation were analogous to what was previously reported for male gametophytes, we provide evidence that *RBR* controls gametic genome duplication, thus genome integrity in the gametophyte-derived progeny. Quantitative reduction of *RBR* in tetraploids led to identification of *rbr* heterozygous plants that displayed novel *RBR* dosage-dependent phenotypes in differentiation and development of the sporophyte albeit the absence of cell cycle defects. These phenotypes coincided with deregulation of conserved epigenetic factors such as Polycomb Repressive Complex 2 (PRC2) genes and *METHYLTRANSFERASE 1* (*MET1*) in the sporophyte, as shown for the gametophytes as well. However, unlike the repression by the PRC2 in gametophytes, *RBR* is activated by the sporophytic PRC2 subunits, suggesting that distinct modules of the conserved *RBR*-PRC2-*MET1* loop control gametophyte and sporophyte generations in plants.

## Introduction

Independent evolution of multicellularity and thus the cell types has implications for adaptation of distinct developmental strategies in plants and animals [1]. Adaptive mechanisms unique to higher plants include alternation between the reduced gametophytic and dominant sporophytic generations, absence of a distinct germ line, and continuous postembryonic development. Unlike animals that develop a germline early in development, the progenitors of gametophytic cell types are derived from sporophytic cells of a mature plant, which acquire competence to undergo meiosis and subsequent mitotic divisions and to establish cell fates of gametic and accessory cell types [2], [3]. Further, double fertilization of gametes leads to the development of an embryo and endosperm. Upon germination, the mature embryo develops into an adult plant by recurrent morphogenetic patterning. Therefore, plant cells must have a flexible but coordinated molecular machinery that helps to maintain their state of competence for cell fate determination and differentiation of distinct cell types during their developmental ontogeny [4]–[6]. In particular, dynamic control of cell fate and differentiation in plants is achieved by regulators of the cell cycle and chromatin complexes in distinct developmental stages, unlike stable gene repression by the same type of regulators during animal development [7], [8].

The tumour suppressor Retinoblastoma (pRB) and closely related proteins are primarily known as negative regulators of the cell cycle and for their antiproliferative activity in multicellular organisms [9], [10]. Specifically, pRB forms a repressive complex with E2F transcription factors to control cell cycle progression from G1 into S phase. Less is known how the pRB pathway functions beyond cell cycle, whether in coordinating cell proliferation and differentiation, or to control early cell fate establishment until late developmental processes [9], [11]. In recent years, pRB homologues have been shown to be necessary in the control of cellular differentiation, stem cell maintenance, and apoptosis in diverse model systems [9], [12], [13] including *Arabidopsis* [14]–[17]. Evolutionary homologues of pRB, either alone or in cooperation with chromatin-associated regulators, can regulate genes involved in cell fate determination and differentiation [9], [13], [18], suggesting a central role of this protein in early cell fate control, as well as subsequent maintenance of the differentiated state and genome integrity [9], [12].

In *Arabidopsis*, *RETINOBLASTOMA RELATED* (*RBR*) is the single homologue of pRB, and the pRB-E2F pathway is largely conserved [7], [9]. Unlike the mouse embryo-lethal pRB knockouts, *Arabidopsis* knock-out alleles of *RBR* are defective in both female and male gametogenesis [14], [15], constraining functional dissection of the pre- and post-gametophytic role of *RBR* in development. Studies that down-regulated *RBR* in distinct tissues using *RBR* RNA interference, virus induced gene silencing or by mis-expression of a RBR-binding viral protein to compete with the native RBR, have not elucidated the genetic behaviour of a *rbr* null mutation during gametophyte or sporophyte development [16], [17], [19]–[21]. In addition, it was unclear in these experiments if both *RBR* mRNA and protein levels were stably reduced throughout development, or aberrantly elevated due to the auto-regulatory function of the pRB-E2F pathway [22]. Nonetheless, these studies have provided an early indication that the *RBR* pathway functions distinctly in different cell types to prevent cell division, endoreduplication and stem cell maintenance. Recent work demonstrated that *RBR* genetically interacts with the conserved epigenetic regulators of the *Polycomb* Repressive Complex 2 (PRC2) to control development of both male and female gametophytes [15], and that *RBR* control of cell fate in the male gametophyte is at least partly coupled to its genetic interaction with the cell cycle associated pollen-specific *CYCLIN-DEPENDENT KINASE A1* (*CDK A1*) [17]. Unlike in the sporophytic leaf, *RBR* is repressed by a maternal and paternal PRC2 complexes during plant reproduction [15], suggesting that the *RBR* regulatory network can function differently depending on the developmental context. Together, the developmental role of *RBR* during sporophytic development remains poorly understood, primarily due to the lack of genetic tools.

In this study, we investigated the effects of an *Arabidopsis RBR* knock-out allele [14], [15] on the plant life cycle. Detailed analysis of *rbr* female gametophytes supported the role of *RBR* in gametophytic cell fate control. Further, we performed a tetraploid genetic analysis that provided direct evidence that at reduced levels of *RBR* sporophyte development is perturbed. When only one out of four functional *RBR* alleles was present in tetraploids triplex for *rbr* (*rbr/rbr/rbr/RBR*), specific stages of sporophytic differentiation and development were affected. The function of *RBR* is therefore partially haplo-insufficient during sporophytic plant development, as revealed by *RBR* dosage analysis in tetraploid plants. Furthermore, we provide genetic evidence that *RBR* functions in concert with the sporophytic PRC2 subunits to control developmental processes in the sporophyte. In short, our work not only illustrates the coordinated function of the RBR pathway in both gametophytes and the sporophyte, it also demonstrates how tetraploid genetics can be exploited to uncover a novel developmental role of an essential regulator during the entire plant life cycle.

## Results/Discussion

### *RBR* is required for cell fate determination in the female gametophyte

**Figure 1:**
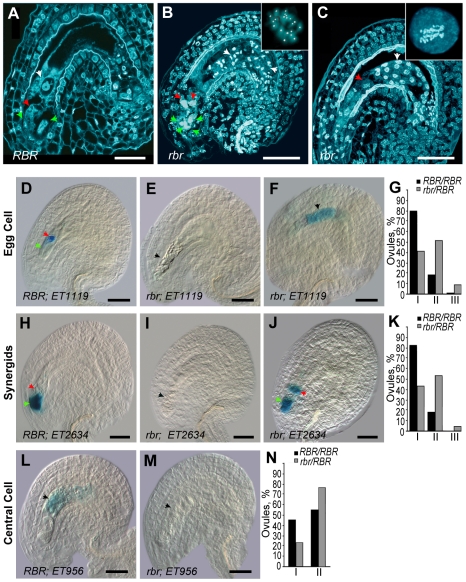
*RBR* is essential for the establishment of cellular identity in the female gametophyte. (A–C) *rbr* embryo sacs (or female gametophytes, FG) continue nuclear proliferation upon cellularization [15] and display ploidy variation. Confocal laser scanning microscopy images of mature ovules 2 days after emasculation [egg cell (red arrow), synergids (green arrows) and central cell nucleus (white arrow)]. Compare the proliferating *rbr* mutant embryo sacs (B, C) to the wild-type embryo sac in (A). In (B), proliferating unfused polar nuclei in the central cell divide synchronously, and they are diploid at anaphase (chromosome number = 10, n = 14 observations; see the inset for a reconstructed image of a representative dividing nucleus). In (C), an egg-cell-like *rbr* nucleus shows endoreduplication, as evident from an excess of metaphase chromosomes (see enlarged image in the inset). (D–N) Cellular identity of the egg cell (D), synergid (H) and central cell (L) are either lost (E, I, M) or deregulated (F, J) in *rbr* embryo sacs. See the text for details. (G, K, N) Histograms of FG phenotypes: class I – ovules with GUS staining in the egg/synergids/central cell as shown in D, H and L, respectively; class II – absence of GUS staining (as shown in E, I and M); and III – GUS mis-expression (deviating from the wild-type patterns as shown in F and J). Total counts for *RBR/RBR* and *rbr/RBR* ovules were 224 and 180, 196 and 208 and 207 and 541 in (G), (K) and (N), respectively. Scale bars: 30 µm.

In *Arabidopsis*, the fully differentiated female gametophyte (embryo sac) consists of only four cell types of clonal origin [3]: a haploid egg cell, a homo-diploid central cell derived from the fusion of two haploid polar nuclei, two synergids that facilitate entry of sperm cells into the embryo sac (Figure 1A), and three antipodal cells that undergo early apoptosis. By characterizing one of the *RBR* knock-out alleles, *rbr-3* [14], we could identify that loss of *RBR* function did not affect the mitotic divisions and cellularization in the female gametophyte [15]. In the majority of cases, however, all cell types including the central cell with unfused polar nuclei commenced proliferation in this mutant (Figure 1B and 1C). The morphological identity of the proliferating *rbr-3* cell types was previously assigned based on their positional information within the embryo sac; however, their molecular identity remained questionable. Therefore, we examined the fate of specific cell types in the absence of *RBR* using cell type-specific molecular markers that are characteristic for the three cell types of the mature female gametophyte. The marker lines *ET1119*, *ET2634*, and *ET956* express β-glucuronidase (GUS) in the egg cell, synergid cells, and the central cell, respectively [23], [24] (Figure 1D, 1H, 1L). In most proliferating *rbr* embryo sacs we could not detect GUS expression in the egg, synergid, and central cell (Figure 1E, 1G, 1I, 1K, 1M, 1N). In 3–8% of the cells, cell type-specific markers showed ectopic expression that deviated from their wild-type pattern (Figure 1F, 1G, 1J, 1K; Figure S1). These findings were further substantiated by loss of gene expression in *rbr* embryo sacs for central cell-specific *FERTILIZATION INDEPENDENT SEED2* (*FIS2*) [15], [25] and for two additional unpublished egg cell-specific genes (A.J. Johnston, H. Bäumlein, T. Dresselhaus, U. Grossniklaus and W. Gruissem, data not shown). Therefore, *RBR* is required for the identity establishment of these gametophytic cell types. In the rare cases where these markers were still present, possibly due to some *RBR* activity carried over from the *rbr*/*RBR* heterozygous megaspore mother cell, they were mis-expressed in the spatial domains of other cell types (Figure 1F and 1J; Figure S1B, S1C, S1D, S1F). For instance, an egg cell marker and a synergid marker were expressed in the central cell and egg cell domains, respectively, in the absence of *RBR*. Therefore, *RBR* not only promotes cell differentiation but also seems to coordinate certain positional information in the female gametophyte.

In a previous work we have shown that the lack of cell differentiation in *rbr* gametophytes paralleled deregulation of certain PRC2 genes and *MET1* [15], whose functional orthologues were known for their role in cell specification, differentiation and also cell cycle regulation in diverse animal systems [26]–[29]. Both our present work and a recent report [25] have established that cell fate, cell cycle and ploidy are also impaired in certain *RBR*-deficient female and male gametophytic cells. There is evidence that RBR directly interacts with MULTICOPYSUPPRESSOR OF IRA1 (MSI1) and FERTILIZATION INDEPENDENT ENDOSPERM (FIE) proteins, which are members of distinct PRC2 complexes in plants [25], [30], [31]. This is consistent with the findings that central cells in *rbr*, *msi1* and *fie* mutant female gametophytes aberrantly proliferate and they are defective either in acquiring cellular identity and/or in heterochromatin status [15], [25]. The phenotypes in *rbr* mutant gametophytes can be partly attributed to the derepression of *MET1* [15], [25], which in turn might result in aberrant hypermethylation, heterochromatin maintenance and/or histone turn over. Interestingly, some of these maternal mutant phenotypes including the defective central cell fate in *rbr* and *msi1* could be rescued by suppressing *MET1* and associated global methylation, suggesting a complex epigenetic control of development [25], [32]. Taken together, it is possible that the *RBR-*PRC2-*MET1* network controls cell fate determination either independently, by co-regulating cell cycle activity, and/or by forming a repressive chromatin modifying complex both in male and female gametophytes.

### *RBR* plays a prominent role in maintaining ploidy and genome integrity

Evolutionary homologues of pRB in animal systems have been implicated in the control of ploidy and chromosomal stability [11]. For instance, pRB-deficient tumors are reported to have elevated aberrant ploidy levels, most likely due to the deregulation of mitotic cell cycle [33], [34]. In *Arabidopsis*, impairment of the RBR-E2F pathway by ectopic expression of the viral RepA protein [19] increased the endocycles in leaf cells. Similar results were obtained when the RBR pathway was perturbed by over-expressing a D3-type cyclin [35], [36] or E2F/DP transcription factors [7]. In all these cases, however, it remained unclear if the ploidy changes were the primary effect of loss or reduction of RBR function. Therefore, we investigated if reduced or loss of *RBR* function in a genetically tractable *rbr* knock-out allele would change developmentally controlled ploidy. Analysis of cellular ploidy in *RBR*-deficient female gametophytic cell types is difficult due to the problems in isolating these miniature cells from plants that are heterozygous for *rbr*. During the morphological analysis of diploid *rbr/RBR* plants using Nomarski optics [15], we noticed that in many instances *rbr* gametophytic nuclei and, in particular, proliferating nuclei in the central cell region were of unusual size. Therefore, we analysed the ploidy of these nuclei by confocal microscopy and subsequent 3D reconstruction of acquired image stacks. We noticed that several *rbr* supernumerary nuclei derived from the unfused polar nuclei had a diploid rather than haploid chromosome number (Figure 1B; 14 observations). This might be due to endoreduplication events in the absence of *RBR* activity [19], as illustrated by an egg-like cell in the inset of Figure 1C as well, where a large excess of metaphase chromosomes was observed. Given that wild-type polar nuclei are haploid [3] and that *rbr* polar nuclei do not fuse to form a homo-diploid central cell [15], it is likely that *RBR* restricts not only ectopic divisions but also polyploidization of haploid polar nuclei. Thus, confocal analysis of the ovules allowed us to demonstrate that absence of *RBR* leads to events of elevated cellular ploidy in the female gametophyte.

Since *RBR* seems to control ploidy of the female gamete(s), this led us to investigate potential changes in plant genome ploidy in *rbr* gamete(s)-derived progeny. We had previously shown that a selfed diploid *rbr/RBR* plant (also referred to as *rbr* mutant) produced viable progeny segregating for wild-type *RBR/RBR* and mutant *rbr/RBR* genotypes, while the female gametophytically lethal *rbr* allele was not transmitted to the next generation [14]. Therefore, the observed rare polyploid egg cells (Figure 1C) might not produce an offspring. If the *rbr* mutant produced viable male gametes with altered ploidy, we would expect that the ploidy of a subset of *rbr* progeny would be different from the parent plant. Indeed, we found that selfed diploid *rbr/RBR* plants produced 6% triploids among *rbr* mutant offspring (n = 56), which produced an array of aneuploid, diploid, and tetraploid plants in the next generation (Figure S2). This phenomenon is normally not observed in diploid wild-type *Arabidopsis*. It is most likely that these triploid progeny resulted from the fusion of a haploid *RBR* egg cell with either a diploid *rbr* sperm cell or two haploid *rbr* sperm cells [14]. Unfortunately, we are unable to test these hypotheses in detail because (i) the chance occurrence of these events was estimated to be 0.1×0.06 = 0.006 considering the *rbr* transmission efficiency of 0.1 and the presence of 6% triploids among the transmitted mutant progeny, and (ii) *rbr* knock-out male gametophytes rarely formed sperm cells [15], [17]. Together, our results suggest that *RBR* controls ploidy maintenance in the gametophytic cells and that it is involved in maintaining genome integrity because in its absence or down-regulation polyploid offspring are produced.

Genome-wide polyploidization has played essential role in speciation and thus evolution of plants [37], however, the factors leading to increased ploidy in plants are not completely understood [38], [39]. Plant autopolyploidization can be preceded by changes in ploidy either somatically, during meiosis, or during male or female gametogenesis. In case of meiosis, asynaptic mutations and meiotic restitution might lead to formation of unreduced gametes and therefore autopolyploids [40]. Thus far, three *Arabidopsis* meiotic mutants, *dyad* [41], *mitosis instead of meiosis* (*mime*) [42] and *jason* [43], were reported to produce unreduced diploid instead of normal haploid gametophytes. Ploidy alterations in female gametogenesis is partly controlled by the maize *INDETERMINATE GAMETOPHYTE 1* (*IG1*) which encodes for a gene with high similarity to *ASYMMETRIC LEAVES2* (*AS2*) in *Arabidopsis* [44]. The *rbr* mutation we report here is the first case in *Arabidopsis* in which aberrations in gametogenesis could result in triploid offspring due to doubling of haploid gametic genome. A “triploid bridge” leading to production of diploids, aneuploids and tetraploids may act as a transition between diploids and autotetraploids and therefore could play a significant role in polyploidization [39], [45]. An induction of triploid offspring as observed in *rbr* knock-out mutants may also occur in the wild-type, should *RBR* activity be altered by unknown environmental factors. Thus, *RBR* might have played a crucial role in plant evolution by controlling genome duplication events.

### *rbr* mutation is recessive in the gametophytes

The roles we propose for wild-type *RBR* in female gametophytic cell specification and differentiation as well as in maintaining genome integrity are only valid if the *rbr-3* allele is gametophytically recessive and genetic reduction of *RBR* function had caused the observed effects. Since *rbr-3* carries a T-DNA insertion in the middle of the *RBR* gene [14], it might generate a truncated protein with a dominant effect. In order to understand the genetic behaviour of *rbr* in the gametophyte, we subjected the gametophytically lethal *rbr* mutation to tetraploid genetic analysis. We asked if the *rbr-3* mutation behaves recessive or dominant in diploid gametophytes produced by tetraploid plants by analysing seed set phenotypes and segregation of *rbr* genotypes in the progeny (Figure S3; see Table 1 and the Materials and Methods section for details). An autonomously tetraploidized plant that was heterozygous for the *rbr* mutation (Figure S2) was subjected to a detailed progeny test (n = 103), which identified three distinct *rbr* genotypes. Seed set and progeny segregation phenotypes of *rbr* simplex (*rbr/RBR/RBR/RBR*), duplex (*rbr/rbr/RBR/RBR*) and triplex (*rbr/rbr/rbr/RBR*) mutant plants significantly fit the recessive model of *rbr-3* inheritance (χ^2^ test; p = 0.05), implicating abortion of homozygous *rbr/rbr* gametophytes and *rbr/RBR* gametophytes giving viable seeds (Table 1).

**Table 1:** Tetraploid genetic analysis by χ^2^-test reveals recessiveness of *rbr-3* allele in gametophyte development.

Genetic models (genotypes)	Seed set (infertile ovules∶developing seeds)			Progeny segregation (R∶S plants^a^)		
	Expected	Observed	χ^2^	Expected	Observed	χ^2^
(a) determination of recessiveness or dominance of the *rbr* allele in tetraploid *rbr* plants						
Simplex, recessive (*rbr/RBR/RBR/RBR*)	28∶650	171∶507	752.69	295∶57	327∶25	21.02
Simplex, dominant (*rbr^D^/RBR/RBR/RBR*)	311∶367	171∶507	116.02	27∶325	327∶25	3543.77
**Duplex, recessive (** ***rbr/rbr/RBR/RBR*** **)**	**151∶527**	**171∶507**	**3.53** *	**324∶28**	**327∶25**	**0.34** *
Duplex, dominant (*rbr^D^/rbr^D^/RBR/RBR*)	527∶151	171∶507	1083.52	91∶261	327∶25	822.10
Triplex, recessive (*rbr/rbr/rbr/RBR*)	367∶311	171∶507	228.81	349∶3	327∶25	194.27
Triplex, dominant (*rbr^D^/rbr^D^/rbr^D^/RBR*)	650∶28	171∶507	8466.08	245∶107	327∶25	89.71
(b) confirmation of recessiveness of the *rbr* allele in simplex and triplex *rbr* plants						
**Simplex, recessive (** ***rbr/RBR/RBR/RBR*** **)**	**11∶255**	**16∶250**	**2.27** *	**305∶102** b	**321∶86**	**3.25** *
**Triplex, recessive (** ***rbr/rbr/rbr/RBR*** **)**	**293∶247**	**309∶232**	**1.89** *	**206∶2**	**208:0**	**1.71** *

**a **Resistance (R) or sensitivity (S) to sulfadiazine (T-DNA selection marker) on MS plates.

**b** Model calculated without considering double reduction.

* χ^2^ value is significant at p = 0.05.

Shown are the phenotypic data of representative individual genotypes. In (a) one tetraploid phenotypic group was tested for all 6 different models (see Figure S2A, S2B for details); in (b) we applied the recessiveness model to two other tetraploid phenotypic groups. The bold font indicates the best fitting model. Note that tetraploid Col wild-type (identified from the segregating tetraploid *rbr* population) had seed set similar to that in diploid Col.

As an additional step to confirm this genetic model, we examined cytological phenotypes of female gametophytes (FG) in these plants. The majority of FGs in both diploid and tetraploid wild-type plants were at stage FG7 upon emasculation, which is typical for wild-type *Arabidopsis* [15]. We noted that all *rbr* embryo sacs in a heterozygous *rbr/RBR* diploid plant showed nuclei proliferation, which significantly fit the expected ratio (χ^2^ = 0.20, p = 0.65, n = 194) as reported previously [14], [15] (Figure S3). In case of the triplex *rbr/rbr/rbr/RBR* plant, three types of FG genotypes are expected: *rbr/rbr, rbr/RBR* and rare *RBR/RBR*. Given that the *rbr* mutation fits a recessive model of inheritance based on the tetraploid seed set phenotype and progeny test (Table 1), only those FGs that had completely lost *RBR* function (*rbr/rbr*) would be expected to show ectopic nuclear divisions, accounting for 54% proliferating FGs (Figure S2). The observed numbers of embryo sacs with supernumerary nuclei in the triplex plants matched the expectation for proliferation of *rbr/rbr* embryo sacs (χ^2^ = 0.076, p = 0.78, n = 162). These data suggest that the viable *rbr/RBR* female gametophytes are likely phenotypically and functionally identical to *RBR/RBR* FGs of wild-type tetraploids, and *RBR* FGs of wild-type diploids.

Together, two independent genetic experiments of seed set and transmission analysis (Table 1) and quantitative analysis of FGs (Figure S3) confirmed that the *rbr* mutation behaves recessive in the female (and male) gametophyte(s). Therefore, we can rule out a dominant (negative or positive) effect of a possible truncated version of *RBR* mRNA or of RBR protein. This situation is perhaps similar to previous reports that premature termination in mouse Rb exons resulted in truncated non-functional proteins [46], [47]. Hence, we conclude that the *rbr-3* allele is a clear loss-of-function mutation of *RBR*.

### Sporophytic development requires dosage-sensitive function of *RBR*

**Figure 2:**
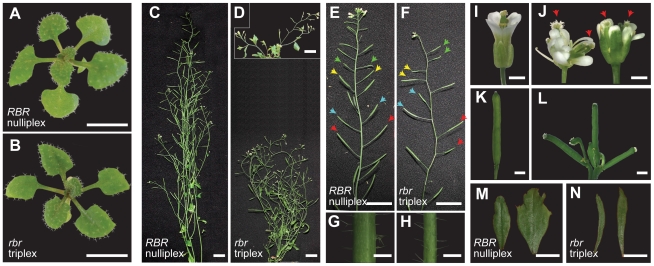
Coordinated plant development requires dosage-dependent function of *RBR*. (A, B) Growth and morphology of a *rbr* triplex seedling (*rbr/rbr/rbr/RBR*) grown on plates for three weeks are indistinguishable from the corresponding tetraploid wild-type nulliplex (*RBR/RBR/RBR/RBR*) in terms of general growth and morphological features including trichome specification. (C–N) Reduction of *RBR* in a triplex *rbr* plant showing strong pleiotropic sporophytic mutant phenotypes only six weeks after planting. Inset in (D): An *rbr* triplex plant with the apparent stunted growth phenotype around the 5^th^ week of planting. (C–F) *rbr* triplex plants showed stunted growth and aberrant plant architecture (D), abnormal phyllotaxy indicated by arrow-head pairs of the same colour (F) [compare to wild-type in (E)], reduced stem thickness (H) and aberrant leaf size and shape (N) in the second and third cauline leaves, in comparison to the wild-type nulliplex (C, E, G, and M, respectively). (J, L) Note that in rare cases, ectopic floral organs were present in some terminal *rbr* triplex flowers (J, red arrows) and multiple terminal young siliques (L), in comparison to the corresponding wild-type (I and K). Scale bars in (A, B) 1 cm, (B–F) 2 cm, (G, H, K, L) 1 mm, (I, J) 1 cm.

**Figure 3:**
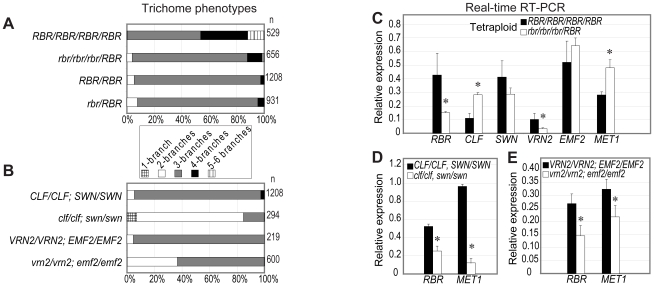
A sporophytic *RBR*–PRC2 regulatory loop mediates trichome differentiation. (A) Trichome branching is impaired when *RBR* is genetically reduced to acute levels (25%) in tetraploids. (B) When the activity of PRC2 genes are either lost (*clf;swn*) or reduced (*vrn2;emf2*) in diploid mutants, trichome branching is affected, in comparison to the corresponding wild-type backgrounds (Ws×Col, L*er*×Col, respectively). (C) Strong reduction of *RBR* alters expression of *CLF* and *VRN2* in tetraploid, suggesting a dosage-dependent gene regulation by *RBR*. Note that the quantitative expression of these genes did not vary between *rbr/RBR* and the wild-type *RBR/RBR* diploid plants (not shown). (D, E) *RBR* is downregulated when sporophytic PRC2 activity is impaired in PRC2 mutants. *significantly different in the mutant when compared to the corresponding wild-type tissues (p = 0.05).

The male and female gametophytic lethality of the *rbr* mutation constrains analysis of *RBR* function during sporophytic development. Tetraploid analysis is therefore an excellent approach to investigate a dosage dependent function of *RBR* in the sporophyte. We recovered and analysed *rbr* tetraploid plants with different numbers of *rbr-3* alleles by large-scale genotyping and segregation analysis of the tetraploid mutant progeny (see Figure 2, Figure S2A and S2B, Table 1). No homozygous tetraploid *rbr* quadruplex (*rbr/rbr/rbr/rbr*) genotypes could be identified, confirming the genetic data (see previous section) that they do not survive post-gametophytically. Specifically, we identified second generation *rbr* triplex plants (*rbr/rbr/rbr/RBR*) that showed significant quantitative reduction of *RBR* expression levels in leaves when compared to the wild-type tetraploids (nulliplex *RBR/RBR/RBR/RBR*) (Figure 3C) or heterozygous diploids (data not shown). A subsequent independent expression analysis reconfirmed that the third generation *rbr* triplex plants also maintained significantly lower *RBR* expression levels in a *RBR* dosage-dependent manner (Figure S4).

We expected that the *rbr* triplex plants, which displayed nearly 75% reduction in gene expression compared to the wild type (Figure S4), could reveal quantitative effects of *RBR* function more readily than diploid *rbr/RBR* plants. Wild-type *RBR/RBR* and heterozygous *rbr/RBR* plants did not differ in sporophytic phenotypes from germination until maturity, indicating full functionality of a single wild-type *RBR* copy (haplo-sufficiency) at the diploid level. Although *rbr* triplex plants appeared to grow normally during early sporophyte development (Figure 2A versus Figure 2B), they showed several developmental phenotypes such as stunted growth habit, aberrant leaf size, altered phyllotaxy of siliques, reduced stem thickness, reduced apical dominance, and ectopic floral organs when they were about six weeks old (Figure 2C–2N). In comparison, the growth and development of *rbr* nulliplex (Figure 2C–2N), simplex and duplex *rbr* plants (not shown) were normal and indistinguishable at similar stages. Thus, the mutant sporophytic phenotypes only occur in *rbr* triplex plants, indicating that a single *RBR* copy cannot sustain normal growth and development and, therefore, *RBR* function is haplo-insufficient in the tetraploid context.

While we anticipated that reduction of *RBR* would alter cell division and/or cell size as was reported from other systems [9], [20], organization and size of cells on the abaxial side of cauline leaves in the triplex mutant surprisingly did not deviate from the corresponding wild-type in young and mature leaves (Figure S5). As *rbr* gametophytes showed ploidy aberrations (as discussed before), we anticipated that ploidy deregulation could also be observed in the leaf sporophyte with decreased *RBR* dosage. However, neither diploid (*rbr/RBR* versus *RBR/RBR*) (data not shown) nor tetraploid (*rbr/rbr/rbr/RBR* versus *RBR/RBR/RBR/RBR*) plants (Figure S6) had significant changes in leaf ploidy when analyzed by flow cytometry. Our genetic results are in contrast to earlier reports that deregulation of *RBR* had immediate consequences on cell divisions and endocycles during leaf organogenesis [19], [21]. We reason that retaining one functional *RBR* copy in diploid and tetraploid systems is sufficient to coordinate cell cycle and specification in the leaf sporophyte and that RBR reduction using a viral RBR-binding protein or virus-induced gene silencing may affect specific functions of RBR that are required for the control of DNA endoreduplication.

Next we asked if differentiation of specific cell types was altered in response to *RBR* dosage change. We examined trichome differentiation patterns in young rosette leaves around 15 days after germination on plates, in diploid and tetraploid plants. As expected, development of trichomes in the 3^rd^ and 4^th^ rosette leaves of diploid *rbr/RBR* plants did not differ from the corresponding wild-type, confirming haplo-sufficiency of *RBR* in diploids (Figure 3A). In wild-type tetraploid plants, over 53% of the trichomes had three branches, 34% had four branches and 12% with 5–6 branches (Figure 3A). These data are consistent with the increased DNA content and supernumerary branching in tetraploids as previously reported [5]. Concomitant with a reduction of *RBR* dosage in *rbr* triplex plants, however, there was a significant reduction of 4-branched (11%) and 5–6 branched trichomes (1%) along with an increase of less-differentiated 3-branched trichomes (84%) (Figure 3A). In addition, we observed a similar trend in *RBR* dosage-dependent reduction of 4-branched trichomes in an independent experiment (Figure S4). Therefore, the single copy of *RBR* in *rbr* triplex is sufficient to specify the trichome cells (Figure 2B versus Figure 2A) but not sufficient to complete full differentiation of this specialized cell type.

It has been proposed that key cell cycle genes that control ploidy restrict trichome branching [5]. Previous studies of down-regulating *RBR* in diploid leaves provided inconclusive results for ploidy-dependent leaf and trichome differentiation. For example, suppression of *RBR* in *Brassica napus* led to elevated ploidy levels in leaves and retarded leaf and trichome development [21]. In contrast, over-expression of a RBR-binding geminivirus RepA protein in diploid *Arabidopsis* in order to interfere with RBR function revealed only marginal elevation of ploidy levels in mature leaves and supernumerary trichome branching patterns [19]. It is unclear, however, if the RepA protein reduced the endogenous *RBR* levels in these plants, or if the transcription of *RBR* was aberrantly elevated due to the autoregulatory function of RBR-E2F pathway [22]. We therefore asked if in single cell trichomes lower RBR levels had caused a concomitant reduction in DNA ploidy, which could explain the fewer branches. By measuring the relative DNA content of individual trichome nuclei by fluorescence microscopy, we found that DNA ploidy in *rbr* triplex trichome cells was comparable to corresponding tetraploid wild type, and that there was no significant difference within ploidy groups across different genotypes (Figure S6). Thus, we conclude that cellular differentiation and morphogenesis of trichomes were affected in a *RBR* dosage-sensitive manner (Figure 3A). Retaining 25% *RBR* in the triplex (*rbr/rbr/rbr/RBR*) plants does not alter the general leaf, trichome and plant ploidy, cell proliferation and trichome specification, but it appears to be insufficient to complete a full differentiation program. This could be particularly true for trichome differentiation, as is also suggested by a recent report that *RBR* is a target of the trichome cell specification and differentiation factors GLABRA1 and GLABRA3 [48]. Obtaining homozygous *rbr* trichomes by inducible methods will be required to analyze how *RBR* controls early specification and/or differentiation. Taken together, the observed sporophytic developmental anomalies including retarded trichome differentiation are a consequence of partial haplo-insufficiency of *RBR* in tetraploids, but not due to *RBR*-mediated cell cycle deregulation.

### *RBR* expression is dynamically regulated consistent with its function in gametophyte and sporophyte development

**Figure 4:**
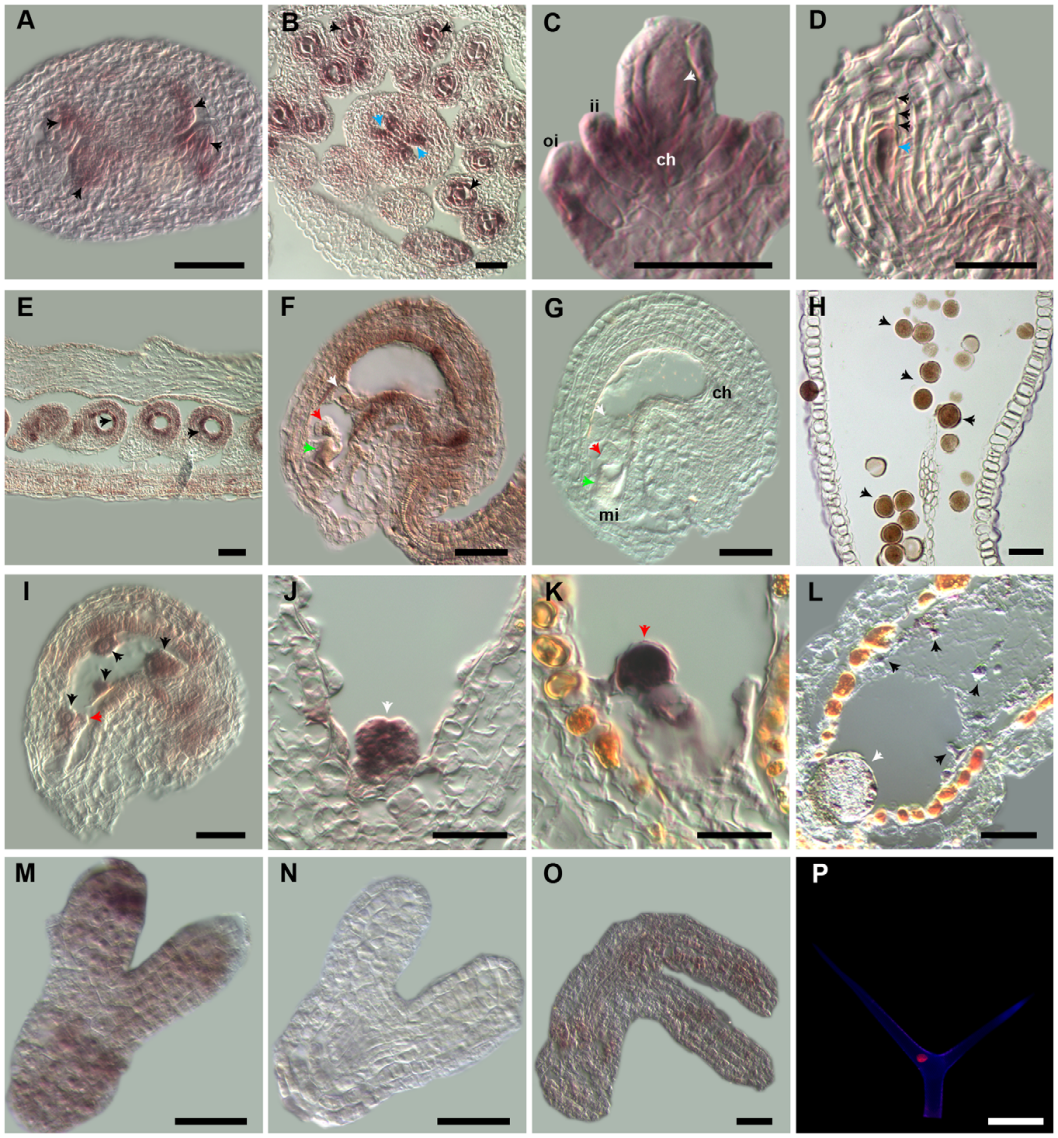
*RBR* is expressed during gametophytic and sporophytic development. Gene expression of *RBR* is determined by mRNA *in situ* hybridization in reproductive tissues (A–O) and by RBR fusion protein (RBR::RFP) analysis in trichomes (P). Black arrows mark archesporial cells (A), microspores (B), degenerating megaspores (D), endothelium (E), mature pollen (H), and free-nuclear endosperm (I, L) at early stages and late stages, respectively. Arrows indicate nucellus (blue arrow, B), megaspore mother cell (white arrow, C), functional megaspore (blue arrow, D), early and late chalazal endosperm (white arrows, J and L), respectively. In (F, G), white, red, green arrows mark the central cell, egg cell and synergids, respectively. Shown in (G, N) are sense controls for corresponding stages in (F, M), respectively. A red arrow in (K) points to a globular embryo. Nuclear localized RBR::RFP fusion protein (white arrow) visualized as red fluorescence (P). ch – chalaza; oi and ii – outer and inner integuments; mi – micropyle. Scale bars in (A–K) and (M–N): 30 µm; in (L): 150 µm; in (O): 40 µm; in (P): 100 µm.

*RBR* is an essential cell cycle regulatory gene that is expressed in the sporophyte (embryo, leaves, root and shoot meristems) and the ovule including the embryo sac [14]–[16], [49]. To gain better insight into the dynamic expression pattern of *RBR* throughout the plant life cycle, we analysed *RBR* RNA and protein accumulation by *in situ* hybridization and a transgenic RBR protein reporter line *RBR::RFP*, respectively [49]. We observed *RBR::RFP* expression throughout sporophyte development in leaves and seedlings (not shown), also during trichome development (Figure 4P). In reproductive tissues, *RBR* mRNA was detected in developing ovules and anthers (Figure 4A and 4B). In particular, *RBR* expression was detected in the functional megaspore, the progenitor cell type of the female gametophyte (Figure 4D). In a fully differentiated embryo sac, we observed *RBR* mRNA expressed in all the embryo sac cell types such as egg cell, central cell and synergid cells, in addition to the sporophytic cells of the ovule (Figure 4F). In contrast, RBR::RFP fusion protein was localized primarily in the central cells [49] suggesting post-translational regulation of RBR in the egg apparatus. A recent study detected RBR::RFP throughout the male gametophyte development [17]. In summary, expression of *RBR* in all cell types of the gametophytes and the sporophyte, including trichome cells, is consistent with the requirement of RBR for cellular proliferation, cell fate and differentiation of the gametophytic cells, sporophytic development and trichome differentiation.

### *RBR* participates in an epigenetic network to control sporophyte development and trichome differentiation

The dynamic expression of *RBR* throughout plant development, and its cell-cycle inter- and independent functions reported thus far suggests that RBR is also involved in other regulatory networks. Evolutionary homologues of pRB and epigenetic factors such as PRC2 proteins and DNA maintenance methyltransferase (Dnmt1) have essential roles in controlling cell differentiation and development both in plants and animals [8], [9], [29]. In animal systems, it has been established that Enhancer of zeste homolog 2 (Ezh2), a core member of PRC2, recruits DNA methyltransferase 1 (Dnmt1), and the resulting maintenance of DNA methylation facilitates formation of more repressive complexes to control distinct developmental processes [50], [51]. There is evidence that both PRC2 genes and Dnmt1 exert their function in a cell cycle-dependent manner. For instance, several PRC2 members and Dnmt1 homologues seem to be directly repressed by the pRB-E2F complexes in plants and animals [29], [51]. Furthermore, PRC2 dynamically regulates pRB or *RBR* via its inherent H3K27me3 activity and possibly through its continuous association throughout the cell cycle [15], [52], [53]. In *Arabidopsis*, there are three distinct orthologues of Ezh2, namely *CURLY LEAF* (*CLF*), which positively regulates cell size and elongation in the leaf sporophyte; *MEDEA* (*MEA*), which negatively regulates cell proliferation and cell size during seed development; and *SWINGER* (*SWN*), which enhances the function of both *CLF* and *MEA* [8], [54]. Similarly, three orthologues of Supressor of zeste 12 (Suz12), which are known to be associated with cell cycle and cell differentiation in animal systems, exist in the *Arabidopsis* genome. *FERTILIZATION INDEPENDENT SEED 2* (*FIS2*) functions similar to *MEA* during seed development; *VERNALIZATION 2* (*VRN2*) and *EMBRYONIC FLOWER 2* (*EMF2*) are associated with distinct sporophytic pathways [8]. *MET1* is the *Arabidopsis* orthologue of Dnmt1, which is a key target of RBR and a modifier of several PRC2 genes, and it is critical for coordinated cell division, specification and differentiation of the embryos, and also throughout the sporophytic development [15], [55]–[57]. The mechanisms by which pRB, PRC2 and Dnmt1 homologues control cellular differentiation and development are not completely understood in plant and animal systems. We recently reported that *RBR*, several PRC2 genes and *MET1* are co-regulated by a negative feedback mechanism during gametophyte differentiation and development [15]. Here we asked if a similar mechanism exists in the leaf sporophyte as well.

First, we compared the expression levels of sporophytic PRC2 genes and *MET1* in *rbr* triplex and heterozygous diploid *rbr* mutant leaf tissues in relation to their corresponding tetraploid or diploid wild-type tissues. Our initial expression analysis in tetraploids suggested that plant to plant variation in expression was quite high. Therefore, we analyzed leaves from individual plants as independent replicates. Prior to gene expression in tetraploids, we examined the expression of *MET1* and PRC2 genes in the diploid wild type and *rbr/RBR* leaf tissues, but we did not detect significant differences in expression levels (not shown). In contrast, we observed that *CLF* and *MET1* were upregulated and *VRN2* was downregulated in *rbr* triplex leaves when compared to the tetraploid wild type (Figure 3C), suggesting the importance of *RBR* dosage for gene regulation in the tetraploid context. This experiment, however, did not reveal if acute genetic down-regulation of *RBR* below 25% would be required for the deregulation of the PRC2 genes *SWN* and *EMF2*. Together, *RBR* regulates *MET1* and the PRC2 genes *CLF* and *VRN2* during leaf development in a dosage-dependent manner. Given that *RBR*-PRC2-*MET1* regulatory network functions during gametophyte development [15], we conclude that *RBR* control of PRC2 and *MET1* is important throughout the plant life cycle.

**Figure 5:**
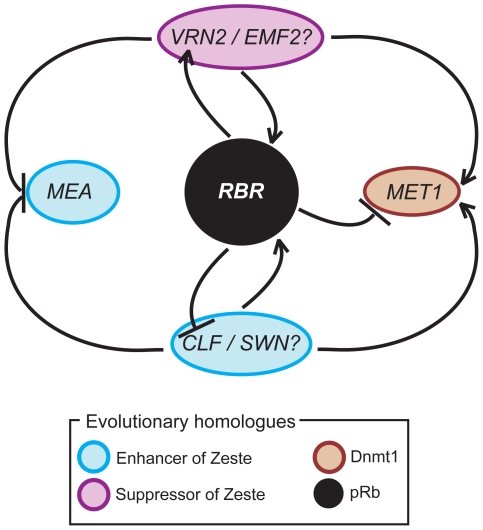
A model showing cross-regulation within *RBR*-PRC2-*MET1* regulatory network in *Arabidopsis* sporophyte. Note that a parallel network operates during male and female gametophyte development [71].

Recent data suggest that *RBR* can function downstream of chromatin regulators like PRC2 or transcription factors such as SCARECROW, GLABRA1 and GLABRA3 during distinct stages of plant development [15], [16], [48]. We therefore asked if PRC2 would reciprocally regulate *RBR* in the leaf sporophyte. We used two different double mutants that disrupted *CLF* and *SWN*, and *VRN2* and *EMF2*, respectively. Sporophytic PRC2 activity is considerably reduced in these double mutants and, consequently, development of the leaf sporophyte is impaired [58]. Although trichomes were correctly specified in the mutant leaves, we observed that their branching was incomplete (Figure 3B). The majority of the trichomes (92–95%) in diploid wild type leaves differentiated to the mature 3-branched stage. The *clf;swn* double mutant showed the most severe phenotype in which the majority of trichomes were 2-branched (78%) while only 15% differentiated to the 3-branch stage. The *vrn2;emf2* double mutant showed similar phenotypes (Figure 3B), although the percentage of trichomes that fully differentiated was higher than that of *clf;swn*, likely because the *emf2* allele used here was a weak loss-of-function allele of *EMF2* [58]. These data collectively suggest that a novel PRC2-dependent epigenetic mechanism operates to control trichome differentiation in addition to leaf development. Intriguingly, expression levels of both *RBR* and *MET1* were significantly reduced in the mutant leaves (Figure 3B, 3D, 3E), suggesting that the sporophytic PRC2 complexes activate both *RBR* and *MET1* in leaves. Previous work demonstrated that *MEA* is derepressed in leaves of PRC2 mutants and that *MEA* is a direct target of the sporophytic PRC2 [59]. Given that MEA represses paternal *RBR* in fertilized female gametophytes [15], it is probable that indirect repression of *RBR* by the sporophytic MEA might have led to reduction of *RBR* levels in PRC2 mutant leaves. Alternatively, reduction of *MET1* in PRC2 mutants is consistent with a previous observation in an animal system that depletion of Ezh2 led to downregulation of Dnmt1 concomitant with local reduction of H3K27me3 activity [60]. Therefore, we propose that both *RBR* and its target gene *MET1* are likely independently activated during leaf development and trichome differentiation either by a cell cycle dependent CLF-PRC2 activity, or indirectly via repression by *MEA*, which in turn is controlled by the CLF-PRC2 (Figure 5). However, due to the complexity of tetraploid wild type and mutant plants used in this study, diploid plants deregulating *RBR* in a temporal and spatial manner will be necessary to test our hypothesis.

### A convergent *RBR*–mediated epigenetic mechanism controls development of the gametophytes and the sporophyte in plants

We have provided here direct evidence that *RBR* has an instructive and dosage-dependent role in cell fate determination, differentiation and development in *Arabidopsis*. This function is partly mediated by a regulatory loop between *RBR* and epigenetic regulators such as PRC2 genes and *MET1*, which operates distinctly in the gametophyte [15] and the sporophyte generations. When *RBR* function is abolished, such as in female or male *rbr* gametophytes [15], [17], proper cell fate assignment does not occur. However, quantitative reduction of *RBR* expression in *rbr* triplex mutant sporophyte does not prevent cell specification but impairs full differentiation, consistent with an earlier study in which stem cell differentiation was delayed when *RBR* was reduced [16]. Since we did not observe significant changes in ploidy levels in response to reduced *RBR* dosage, it is likely that *RBR*-mediated developmental functions can also be cell cycle-independent, similar to pRB control of cell cycle-unrelated processes in animals [18], [61], [62]. In support of the *RBR*-PRC2-*MET1* epigenetic network that we have identified, a recent study revealed that DNA methylation of *RBR*, *CLF*, *SWN*, *VRN2* and *EMF2* loci is regulated by *MET1* in *Arabidopsis* sporophyte [63]. We propose that the dynamic modulation of this *RBR*-PRC2-*MET1* circuit was adopted to accommodate the regulation of distinct developmental processes in both gametophyte and sporophyte generations.

## Materials and Methods

### Plant material and growth conditions

The *rbr-3* allele (Col background) [14], cell-specific marker lines *ET956*, *ET1119* and *ET2634* (L*er* background) [23], [24], *RBR::RFP* reporter line [64], and the PRC2 mutant alleles, *clf-50* (Ws), *swn-3* (Col), *emf2-10* (Ws) and *vrn2-1* (L*er*) [58] were described previously. Ploidy level of mutant plants was determined using a flow-cytometer (Partec GmbH, Munster, Germany). For trichome quantification, plants were germinated on MS plates without sucrose in growth cabinets, and classes of trichomes were counted on the 3^rd^ and 4^th^ leaves, when the seedlings were at 5–6 leaf stage.

### Histological analyses

Confocal analysis of ovules and spatial analysis of GUS activity in ovules and seed tissues were performed as described earlier [6], [15]. Scanning electron micrographs of leaves were prepared as published [65].

### Ploidy measurements in trichomes

Cauline leaves of mutant and wild-type tissues were fixed in a formaldehyde/glutaraldehyde fixative. Intact trichome cells were isolated from leaf epidermis by an established protocol based on removal of Ca^2+^ ions [66]. Nuclear images of guard cells from leaves (n = 42 and 54 in wild type and mutant, respectively), and of the trichome cells of the wild-type (n = 80) and the mutant (n = 42) were recorded for fluorescence measurement upon DAPI staining by confocal microscopy (LSM 510META, Carl Zeiss, Jena, Germany). DAPI was visualized with a 364 nm laser line in combination with a 380–475 nm bandpass filter. Recordings were made with a 20x objective at zoom 4, with maximum pinhole. Fluorescence intensity was analysed with the LSM software (release 3.2). Data normalization with average fluorescence values of the tetraploid guard cell nuclei (4C) and arbitrary clustering of data points were performed as described elsewhere [67].

### RNA *in situ* hybridization

Semi-thin paraffin sections of inflorescences, emasculated pistils, and siliques [68] were used for hybridization with the hydrolyzed digoxygenin-UTP-labeled riboprobes (Roche Diagnostics, Basel, Switzerland) that were prepared using a *RBR* cDNA expression clone as a template. *In situ* hybridization was performed as before [6].

### Tetraploid genetic analysis of *rbr-3*

Tests for dominance/recessiveness were performed as previously described [69]. The expected phenotypic ratios for recessive and dominant genetic models were calculated considering (a) reduced transmission efficiency of the *rbr-3* allele and (b) maximal double reduction. Transmission efficiency (TE) is an estimate of inheritance of a mutant allele versus the wild-type allele by female or male gametes [14]. It is calculated as a ratio of number of mutant plants to wild-type plants in progenies from reciprocal crosses of a heterozygous mutant. The *rbr* allele is not transmitted through female gametes (TE_♀(*rbr*)_ = 0) [14]; the transmission of *rbr* through pollen was estimated as 0.1 based on both TE_♀(*rbr*)_ in diploid condition, and recovery of triplex plants in triplex progeny (as a ratio of triplex to duplex plants). Double reduction describes the situation in polyploids, in which a heterozygous individual produces homozygous gametes [69]. This can occur if quadrivalents are formed and recombination occurs between the centromere and the locus of interest. Through chromatid segregation both alleles of the sister chromatids can co-exist in the same gamete. Thus, the frequency of double reduction depends on the distance between the locus in question and the centromere. Because the *RBR* locus is ∼45 cM away from the centromere, these loci can be considered unlinked. Therefore, we used the maximal double reduction frequency of 1/6 for our calculations (see Text S1).

As we did not know how many *rbr-3* alleles were present in the tetraploid plants, we compared the observed data to six different models with one, two, or three *rbr-3* alleles, them being dominant and recessive, respectively. First we recorded the seed set/sterility phenotypes of a total of 103 progeny plants originating from a selfed autonomously tetraploidized plant, which was heterozygous for the *rbr* mutation (Figure S2A, S2B). Out of the 103 progeny plants, we took one tetraploid plant group (consisting of 52 plants) with similar seed set and subjected the sterility phenotype and progeny segregation data for 6 different models [simplex, recessive (*rbr/RBR/RBR/RBR*); simplex, dominant (*rbr^D^/RBR/RBR/RBR*); duplex, recessive (*rbr/rbr/RBR/RBR*); duplex, dominant (*rbr^D^/rbr^D^/RBR/RBR*); triplex, recessive (*rbr/rbr/rbr/RBR*); triplex, dominant (*rbr^D^/rbr^D^/rbr^D^/RBR*)]. These plants were identified as duplex-recessive for *rbr* (Table 1). Subsequently, two other distinct tetraploid phenotype groups were fit to simplex-recessive and triplex-recessive models (41 and 2 plants, respectively) (see Figure S2 and Table 1 for details). Progeny analysis of one of these two triplex plants identified in this experiment confirmed stability of the seed set phenotype over subsequent generation (Figure S4).

### Quantitative real-time RT–PCR

RNA extraction and reverse transcription were performed as described [15]. Quantitative real-time measurements were performed using SYBR Green Fast Master Mix reagent in an ABI Prism 7500 Sequence Detection System (Applied Biosystems) (Applied Biosystems), according to the manufacturer's instructions. For each condition, 2 technical replicates and 3 biological replicates were used. Relative gene expression levels were normalized to the expression levels of a control gene, *PP2A* (At1g13320) [70]. Primers used in this work are listed in Table S1.

## Supporting Information

Figure S1 Deregulation of cell-specific markers in *rbr* female gametophyte. (A–D) the enhancer detector *ET1119* (egg cell marker) *GUS* expression in wild-type and *rbr* female gametophytes. (A) A wild-type embryo sac at maturity showing a typical egg-specific GUS expression pattern of *ET1119* at 2 days after emasculation (red arrow). Green arrow marks synergids. (B–D) Mis-expression of the egg-specific GUS marker in *rbr* mutant embryo sacs. In some small number of cases, the ectopic GUS staining was restricted to the egg (red arrow) and central cell region (black arrow) (B) (2 observations) or the whole FG (C, black arrow) (1 observation). (D) In this particular *rbr* embryo sac, two big cells in the place of an egg were stained (red arrows) (1 observation). Note that the synergids appeared morphologically normal, but they also showed GUS expression (green arrows). (E) Synergid-specific expression of *ET2634* in the wild-type (green arrow). (F) Rare mis-expression phenotype of *ET2634* in *rbr* embryo sac. Black arrow points to egg apparatus (mainly synergid-derived proliferation) with a weaker GUS. Scale bars: 30 µm.
(2.47 MB TIF)

Figure S2 Schemes of tetraploid genetics dissecting *RBR* function. (A) *RBR* mediated triploid bridge led to autonomous tetraploidization of diploid plants heterozygous for *rbr*. Shown are representative flow cytometry histograms depicting the cellular ploidy of young cauline leaves. (B) Progeny testing (n = 103) of a tetraploid *rbr* heterozygote identified *rbr* triplex plants (shaded in yellow) (see Table 1 for details). (C) A second generation progeny test of an *rbr* triplex plant (n = 93) (see Figure S4 for additional data).
(2.17 MB DOC)

Figure S3 Comparison of female gametophyte phenotypes in *rbr* triplex versus the corresponding tetraploid wild-type confirms gametophytic recessiveness of the *rbr-3* allele. (A) Histogram of female gametophyte (FG) phenotypes in diploid *rbr* plants *(rbr/RBR)* in comparison to the corresponding wild-type (RBR/RBR). In the expected genetic model we considered that FGs homozygous for *rbr* hyper-proliferate and they are lethal, typical of the *rbr* gametophytic lethal mutation [15]. (B) Histogram of female gametophyte (FG) phenotypes in tetraploid triplex plants (*rbr/rbr/rbr/RBR*) in comparison to the tetraploid wild type (*RBR/RBR/RBR/RBR*). The expected ratio of FG phenotypes in the triplex plant was calculated based on a genetic model for recessiveness (in our case full loss of function) considering double reduction (see Table 1). obs: observed FG phenotypes; exp: expected FG phenotypes; class FG7: mature 4-celled wild-type female gametophyte; class Proliferation: FGs with ectopic cell proliferation. Total counts for *RBR/RBR* and *rbr/RBR* ovules were 101 and 194, for *RBR/RBR/RBR/RBR* and *rbr/rbr/rbr/RBR* 108 and 162, respectively.
(0.45 MB TIF)

Figure S4 Quantitative reduction of *RBR* expression and concomitant reduction of the characteristic 4-branched trichomes in tetraploid leaves confirms *RBR* dosage-dependent trichome differentiation. Note that reduction in *RBR* levels ultimately correlated with reduction in seed set. Analyzed are four distinct genotypes, *RBR/RBR/RBR/RBR* (n = 178 and 415, for seed set and trichome counts, respectively); *rbr/RBR/RBR/RBR* (n = 259 and 387); *rbr/rbr/RBR/RBR* (n = 214 and 395); and *rbr/rbr/rbr/RBR* (n = 217 and 432). *significantly different in the mutant when compared to the corresponding nulliplex (p = 0.05).
(0.03 MB DOC)

Figure S5 Loss of three functional copies of RBR in tetraploids does not lead to aberrant cell division and cell size in leaves. Shown are scanning electron micrographs of abaxial region of mature cauline leaves in (A) diploid Col wild-type (*RBR/RBR)*, (B) diploid *rbr* mutant (*RBR/rbr*), (C) tetraploid Col wild-type (*RBR/RBR/RBR/RBR*) and (D) *rbr* triplex (*RBR/rbr/rbr/rbr*). Scale = 90 µm.
(6.86 MB TIF)

Figure S6 Quantitative reduction of *RBR* in tetraploids does not lead to changes in ploidy of leaf and trichome cells. (A) Leaf ploidy in tetraploid wild type (*RBR/RBR/RBR/RBR*) and *rbr* triplex (*rbr/rbr/rbr/RBR*) recorded by flow cytometry (B) Ploidy classes of the trichomes in reference to the tetraploid (4C) guard cells, upon nuclear DNA quantification by confocal microscopy.
(0.51 MB PPT)

Table S1 Primers used in quantitative real time PCR assays.
(0.03 MB DOC)

Text S1 Tetraploid genetics and double reduction.
(0.09 MB DOC)

## References

[1] Meyerowitz EM (2002). Plants compared to animals: the broadest comparative study of development.. Science.

[2] McCormick S (2004). Control of male gametophyte development.. Plant Cell.

[3] Yadegari R, Drews GN (2004). Female gametophyte development.. Plant Cell.

[4] Day RC, Herridge RP, Ambrose BA, Macknight RC (2008). Transcriptome analysis of proliferating *Arabidopsis* endosperm reveals biological implications for the control of syncytial division, cytokinin signaling, and gene expression regulation.. Plant Physiol.

[5] Hulskamp M (2004). Plant trichomes: a model for cell differentiation.. Nat Rev Mol Cell Biol.

[6] Johnston AJ, Meier P, Gheyselinck J, Wuest SE, Federer M (2007). Genetic subtraction profiling identifies genes essential for *Arabidopsis* reproduction and reveals interaction between the female gametophyte and the maternal sporophyte.. Genome Biol.

[7] De Veylder L, Beeckman T, Inze D (2007). The ins and outs of the plant cell cycle.. Nat Rev Mol Cell Biol.

[8] Kohler C, Villar CB (2008). Programming of gene expression by Polycomb group proteins.. Trends Cell Biol.

[9] Gruissem W, Inze D (2007). Function of the Retinoblastoma-related protein in plants.. Cell Cycle Control and Plant Development.

[10] Kaelin WGJ (1999). Functions of the retinoblastoma protein.. Bioessays.

[11] Korenjak M, Brehm A (2006). The retinoblastoma tumour suppressor in model organisms—new insights from flies and worms.. Curr Mol Med.

[12] Knudsen ES, Sexton CR, Mayhew CN (2006). Role of the retinoblastoma tumor suppressor in the maintenance of genome integrity.. Curr Mol Med.

[13] Korenjak M, Brehm A (2005). E2F-Rb complexes regulating transcription of genes important for differentiation and development.. Curr Opin Genet Dev.

[14] Ebel C, Mariconti L, Gruissem W (2004). Plant retinoblastoma homologues control nuclear proliferation in the female gametophyte.. Nature.

[15] Johnston AJ, Matveeva E, Kirioukhova O, Grossniklaus U, Gruissem W (2008). A Dynamic reciprocal RBR-PRC2 regulatory circuit controls *Arabidopsis* gametophyte development.. Curr Biol.

[16] Wildwater M, Campilho A, Perez-Perez JM, Heidstra R, Blilou I (2005). The *RETINOBLASTOMA-RELATED* gene regulates stem cell maintenance in *Arabidopsis* roots.. Cell.

[17] Chen Z, Hafidh S, Poh SH, Twell D, Berger F (2009). Proliferation and cell fate establishment during *Arabidopsis* male gametogenesis depends on the Retinoblastoma protein.. Proc Natl Acad Sci U S A.

[18] Goodrich DW (2006). The retinoblastoma tumor-suppressor gene, the exception that proves the rule.. Oncogene.

[19] Desvoyes B, Ramirez-Parra E, Xie Q, Chua NH, Gutierrez C (2006). Cell type-specific role of the retinoblastoma/E2F pathway during *Arabidopsis* leaf development.. Plant Physiol.

[20] Jordan CV, Shen W, Hanley-Bowdoin LK, Robertson DN (2007). Geminivirus-induced gene silencing of the tobacco retinoblastoma-related gene results in cell death and altered development.. Plant Mol Biol.

[21] Park JA, Ahn JW, Kim YK, Kim SJ, Kim JK (2005). Retinoblastoma protein regulates cell proliferation, differentiation, and endoreduplication in plants.. Plant J.

[22] Park K, Choe J, Osifchin NE, Templeton DJ, Robbins PD (1994). The human retinoblastoma susceptibility gene promoter is positively autoregulated by its own product.. J Biol Chem.

[23] Chen YH, Li HJ, Shi DQ, Yuan L, Liu J (2007). The central cell plays a critical role in pollen tube guidance in *Arabidopsis*.. Plant Cell.

[24] Gross-Hardt R, Kagi C, Baumann N, Moore JM, Baskar R (2007). LACHESIS restricts gametic cell fate in the female gametophyte of *Arabidopsis*.. PLoS Biol.

[25] Jullien PE, Mosquna A, Ingouff M, Sakata T, Ohad N (2008). Retinoblastoma and its binding partner MSI1 control imprinting in *Arabidopsis*.. PLoS Biol.

[26] Creyghton MP, Markoulaki S, Levine SS, Hanna J, Lodato MA (2008). H2AZ is enriched at polycomb complex target genes in ES cells and is necessary for lineage commitment.. Cell.

[27] Pasini D, Bracken AP, Hansen JB, Capillo M, Helin K (2007). The polycomb group protein Suz12 is required for embryonic stem cell differentiation.. Mol Cell Biol.

[28] Shen X, Liu Y, Hsu YJ, Fujiwara Y, Kim J (2008). EZH1 mediates methylation on histone H3 lysine 27 and complements EZH2 in maintaining stem cell identity and executing pluripotency.. Mol Cell.

[29] Ferreira R, Naguibneva I, Pritchard LL, Ait-Si-Ali S, Harel-Bellan A (2001). The Rb/chromatin connection and epigenetic control: opinion.. Oncogene.

[30] Ach RA, Taranto P, Gruissem W (1997). A conserved family of WD-40 proteins binds to the retinoblastoma protein in both plants and animals.. Plant Cell.

[31] Katz A, Oliva M, Mosquna A, Hakim O, Ohad N (2004). FIE and CURLY LEAF polycomb proteins interact in the regulation of homeobox gene expression during sporophyte development.. Plant J.

[32] Kohler C, Makarevich G (2006). Epigenetic mechanisms governing seed development in plants.. EMBO Rep.

[33] Hernando E, Nahle Z, Juan G, Diaz-Rodriguez E, Alaminos M (2004). Rb inactivation promotes genomic instability by uncoupling cell cycle progression from mitotic control.. Nature.

[34] Srinivasan SV, Mayhew CN, Schwemberger S, Zagorski W, Knudsen ES (2007). RB loss promotes aberrant ploidy by deregulating levels and activity of DNA replication factors.. J Biol Chem.

[35] Dewitte W, Riou-Khamlichi C, Scofield S, Healy JM, Jacqmard A (2003). Altered cell cycle distribution, hyperplasia, and inhibited differentiation in *Arabidopsis* caused by the D-type cyclin CYCD3.. Plant Cell.

[36] Dewitte W, Scofield S, Alcasabas AA, Maughan SC, Menges M (2007). *Arabidopsis* CYCD3 D-type cyclins link cell proliferation and endocycles and are rate-limiting for cytokinin responses.. Proc Natl Acad Sci U S A.

[37] Rieseberg LH, Willis JH (2007). Plant speciation.. Science.

[38] Thompson JD, Lumaret R (1992). The evolutionary dynamics of polyploid plants: origins, establishment and persistence.. Trends Ecol Evol.

[39] Henry IM, Dilkes BP, Comai L (2007). Genetic basis for dosage sensitivity in *Arabidopsis thaliana*.. PLoS Genet.

[40] Cai X, Xu SS (2007). Meiosis-driven genome variation in plants.. Curr Genomics.

[41] Ravi M, Marimuthu MP, Siddiqi I (2008). Gamete formation without meiosis in *Arabidopsis*.. Nature.

[42] d'Erfurth I, Jolivet S, Froger N, Catrice O, Novatchkova M (2009). Turning meiosis into mitosis.. PLoS Biol.

[43] Erilova A, Brownfield L, Exner V, Rosa M, Twell D (2009). Imprinting of the polycomb group gene *MEDEA* serves as a ploidy sensor in *Arabidopsis*.. PLoS Genet.

[44] Evans MM (2007). The *indeterminate gametophyte1* gene of maize encodes a LOB domain protein required for embryo sac and leaf development.. Plant Cell.

[45] Burton TL, Husband BC (2000). Fitness differences among diploids, tetraploids, and their triploid progeny in *Chamerion angustifolium*: mechanisms of inviability and implications for polyploid evolution.. Evolution.

[46] Lee EY, Chang CY, Hu N, Wang YC, Lai CC (1992). Mice deficient for Rb are nonviable and show defects in neurogenesis and haematopoiesis.. Nature.

[47] Jacks T, Fazeli A, Schmitt EM, Bronson RT, Goodell MA (1992). Effects of an Rb mutation in the mouse.. Nature.

[48] Morohashi K, Grotewold E (2009). A systems approach reveals regulatory circuitry for *Arabidopsis* trichome initiation by the GL3 and GL1 selectors.. PLoS Genet.

[49] Ingouff M, Hamamura Y, Gourgues M, Higashiyama T, Berger F (2007). Distinct dynamics of HISTONE3 variants between the two fertilization products in plants.. Curr Biol.

[50] Fiskus W, Buckley K, Rao R, Mandawat A, Yang Y (2009). Panobinostat treatment depletes EZH2 and DNMT1 levels and enhances decitabine mediated de-repression of JunB and loss of survival of human acute leukemia cells.. Cancer Biol Ther.

[51] Schwartz YB, Pirrotta V (2007). Polycomb silencing mechanisms and the management of genomic programmes.. Nat Rev Genet.

[52] Kotake Y, Cao R, Viatour P, Sage J, Zhang Y (2007). pRB family proteins are required for H3K27 trimethylation and Polycomb repression complexes binding to and silencing p16INK4alpha tumor suppressor gene.. Genes Dev.

[53] Blais A, van Oevelen CJ, Margueron R, Acosta-Alvear D, Dynlacht BD (2007). Retinoblastoma tumor suppressor protein-dependent methylation of histone H3 lysine 27 is associated with irreversible cell cycle exit.. J Cell Biol.

[54] Kim GT, Tsukaya H, Uchimiya H (1998). The *CURLY LEAF* gene controls both division and elongation of cells during the expansion of the leaf blade in *Arabidopsis thaliana*.. Planta.

[55] Jullien PE, Kinoshita T, Ohad N, Berger F (2006). Maintenance of DNA methylation during the *Arabidopsis* life cycle is essential for parental imprinting.. Plant Cell.

[56] Xiao W, Custard KD, Brown RC, Lemmon BE, Harada JJ (2006). DNA methylation is critical for *Arabidopsis* embryogenesis and seed viability.. Plant Cell.

[57] Finnegan EJ, Peacock WJ, Dennis ES (1996). Reduced DNA methylation in *Arabidopsis thaliana* results in abnormal plant development.. Proc Natl Acad Sci U S A.

[58] Chanvivattana Y, Bishopp A, Schubert D, Stock C, Moon YH (2004). Interaction of Polycomb-group proteins controlling flowering in *Arabidopsis*.. Development.

[59] Jullien PE, Katz A, Oliva M, Ohad N, Berger F (2006). Polycomb group complexes self-regulate imprinting of the Polycomb group gene MEDEA in *Arabidopsis*.. Curr Biol.

[60] Wu X, Gong Y, Yue J, Qiang B, Yuan J (2008). Cooperation between EZH2, NSPc1-mediated histone H2A ubiquitination and Dnmt1 in HOX gene silencing.. Nucleic Acids Res.

[61] Chi W, Reinke V (2006). Promotion of oogenesis and embryogenesis in the *C. elegans* gonad by EFL-1/DPL-1 (E2F) does not require LIN-35 (pRB).. Development.

[62] De Falco G, Comes F, Simone C (2006). pRb: master of differentiation. Coupling irreversible cell cycle withdrawal with induction of muscle-specific transcription.. Oncogene.

[63] Lister R, O'Malley RC, Tonti-Filippini J, Gregory BD, Berry CC (2008). Highly integrated single-base resolution maps of the epigenome in *Arabidopsis*.. Cell.

[64] Ingouff M, Jullien PE, Berger F (2006). The female gametophyte and the endosperm control cell proliferation and differentiation of the seed coat in *Arabidopsis*.. Plant Cell.

[65] Schneitz K, Hulskamp M, Kopczak SD, Pruitt RE (1997). Dissection of sexual organ ontogenesis: a genetic analysis of ovule development in *Arabidopsis thaliana*.. Development.

[66] Zhang X, Oppenheimer DG (2004). A simple and efficient method for isolating trichomes for downstream analyses.. Plant Cell Physiol.

[67] Borghi L, Bureau M, Simon R (2007). *Arabidopsis* JAGGED LATERAL ORGANS is expressed in boundaries and coordinates KNOX and PIN activity.. Plant Cell.

[68] Kerk NM, Ceserani T, Tausta SL, Sussex IM, Nelson TM (2003). Laser capture microdissection of cells from plant tissues.. Plant Physiol.

[69] Burnham CR (1964). Discussions in Cytogenetics..

[70] Czechowski T, Stitt M, Altmann T, Udvardi MK, Scheible WR (2005). Genome-wide identification and testing of superior reference genes for transcript normalization in *Arabidopsis*.. Plant Physiol.

[71] Johnston AJ, Gruissem W (2009). Gametophyte differentiation and imprinting control in plants: Crosstalk between RBR and chromatin.. Commun Integr Biol.

