# Supplementary figures and images for: Dosage-Sensitive Function of RETINOBLASTOMA RELATED and Convergent Epigenetic Control Are Required during the Arabidopsis Life Cycle
Source: PLoS Genet. 2010 Jun 17;6(6):e1000988. doi: 10.1371/journal.pgen.1000988 (PMC2887464; doi:10.1371/journal.pgen.1000988)

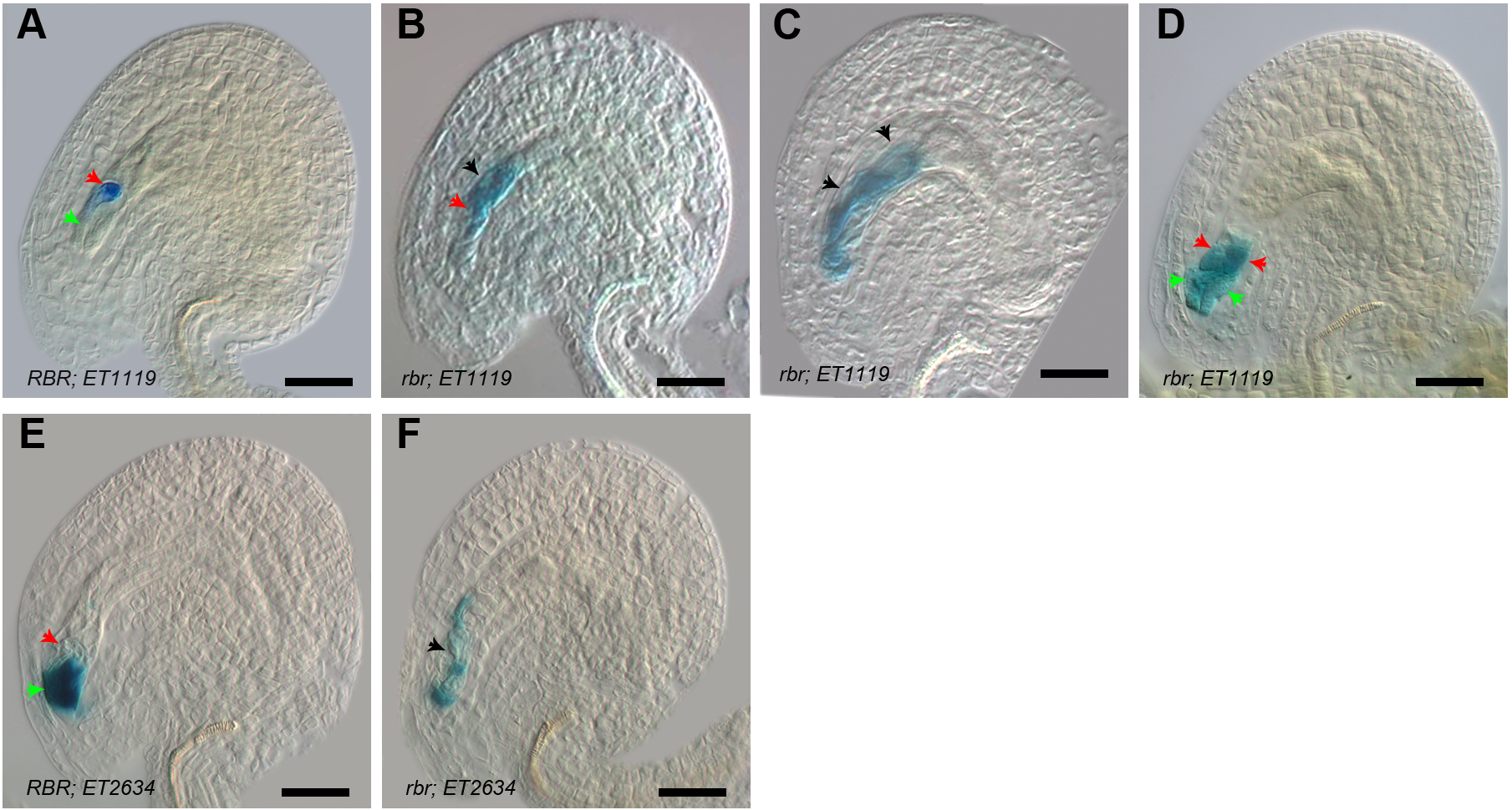

Supplement: Figure S1 — Deregulation of cell-specific markers in rbr female gametophyte. (A–D) the enhancer detector ET1119 (egg cell marker) GUS expression in wild-type and rbr female gametophytes. (A) A wild-type embryo sac at maturity showing a typical egg-specific GUS expression pattern of ET1119 at 2 days after emasculation (red arrow). Green arrow marks synergids. (B–D) Mis-expression of the egg-specific GUS marker in rbr mutant embryo sacs. In some small number of cases, the ectopic GUS staining was restricted to the egg (red arrow) and central cell region (black arrow) (B) (2 observations) or the whole FG (C, black arrow) (1 observation). (D) In this particular rbr embryo sac, two big cells in the place of an egg were stained (red arrows) (1 observation). Note that the synergids appeared morphologically normal, but they also showed GUS expression (green arrows). (E) Synergid-specific expression of ET2634 in the wild-type (green arrow). (F) Rare mis-expression phenotype of ET2634 in rbr embryo sac. Black arrow points to egg apparatus (mainly synergid-derived proliferation) with a weaker GUS. Scale bars: 30 µm. (2.47 MB TIF) [file pgen.1000988.s001.tif]

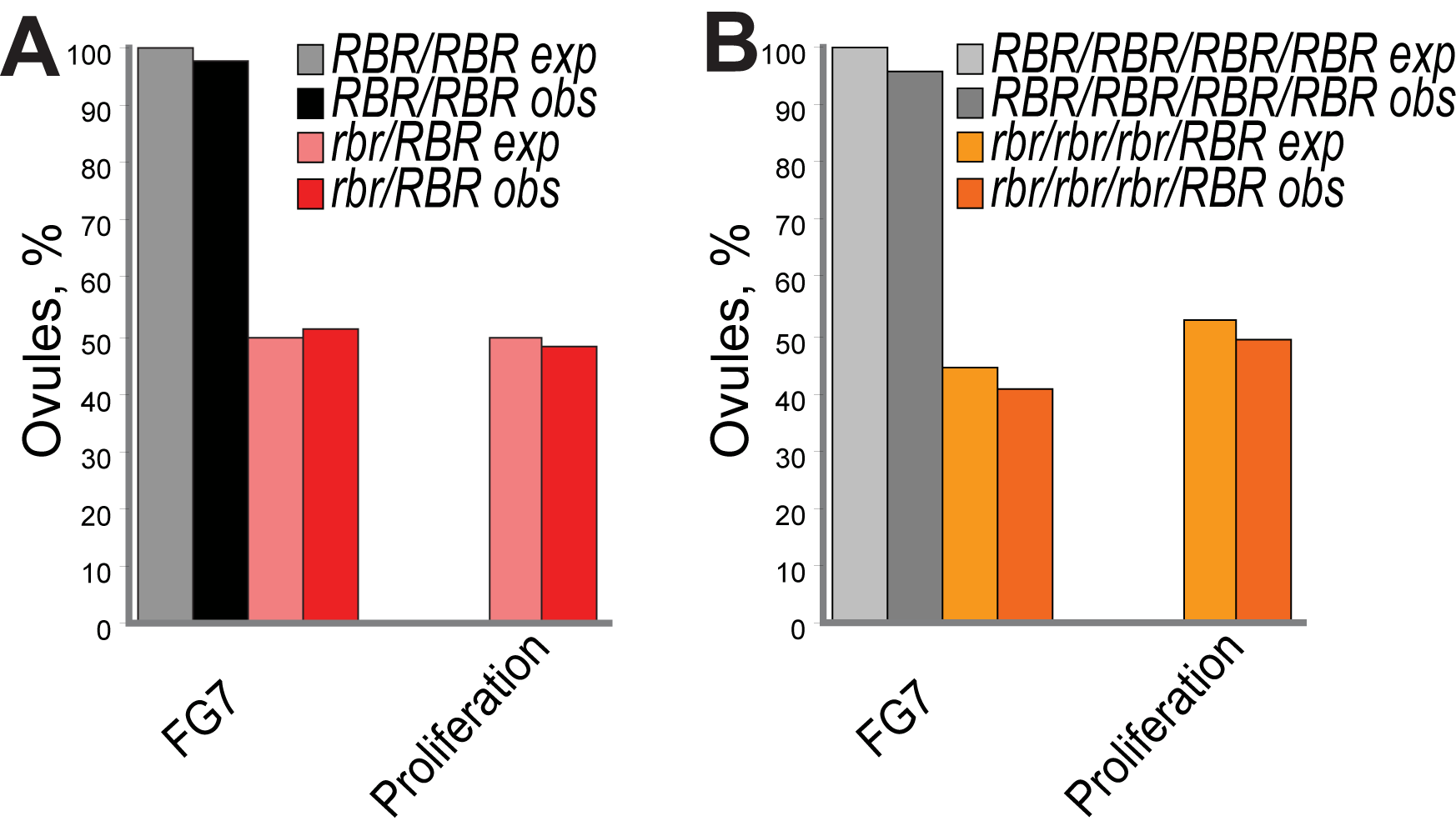

Supplement: Figure S3 — Comparison of female gametophyte phenotypes in rbr triplex versus the corresponding tetraploid wild-type confirms gametophytic recessiveness of the rbr-3 allele. (A) Histogram of female gametophyte (FG) phenotypes in diploid rbr plants (rbr/RBR) in comparison to the corresponding wild-type (RBR/RBR). In the expected genetic model we considered that FGs homozygous for rbr hyper-proliferate and they are lethal, typical of the rbr gametophytic lethal mutation [15]. (B) Histogram of female gametophyte (FG) phenotypes in tetraploid triplex plants (rbr/rbr/rbr/RBR) in comparison to the tetraploid wild type (RBR/RBR/RBR/RBR). The expected ratio of FG phenotypes in the triplex plant was calculated based on a genetic model for recessiveness (in our case full loss of function) considering double reduction (see Table 1). obs: observed FG phenotypes; exp: expected FG phenotypes; class FG7: mature 4-celled wild-type female gametophyte; class Proliferation: FGs with ectopic cell proliferation. Total counts for RBR/RBR and rbr/RBR ovules were 101 and 194, for RBR/RBR/RBR/RBR and rbr/rbr/rbr/RBR 108 and 162, respectively. (0.45 MB TIF) [file pgen.1000988.s003.tif]

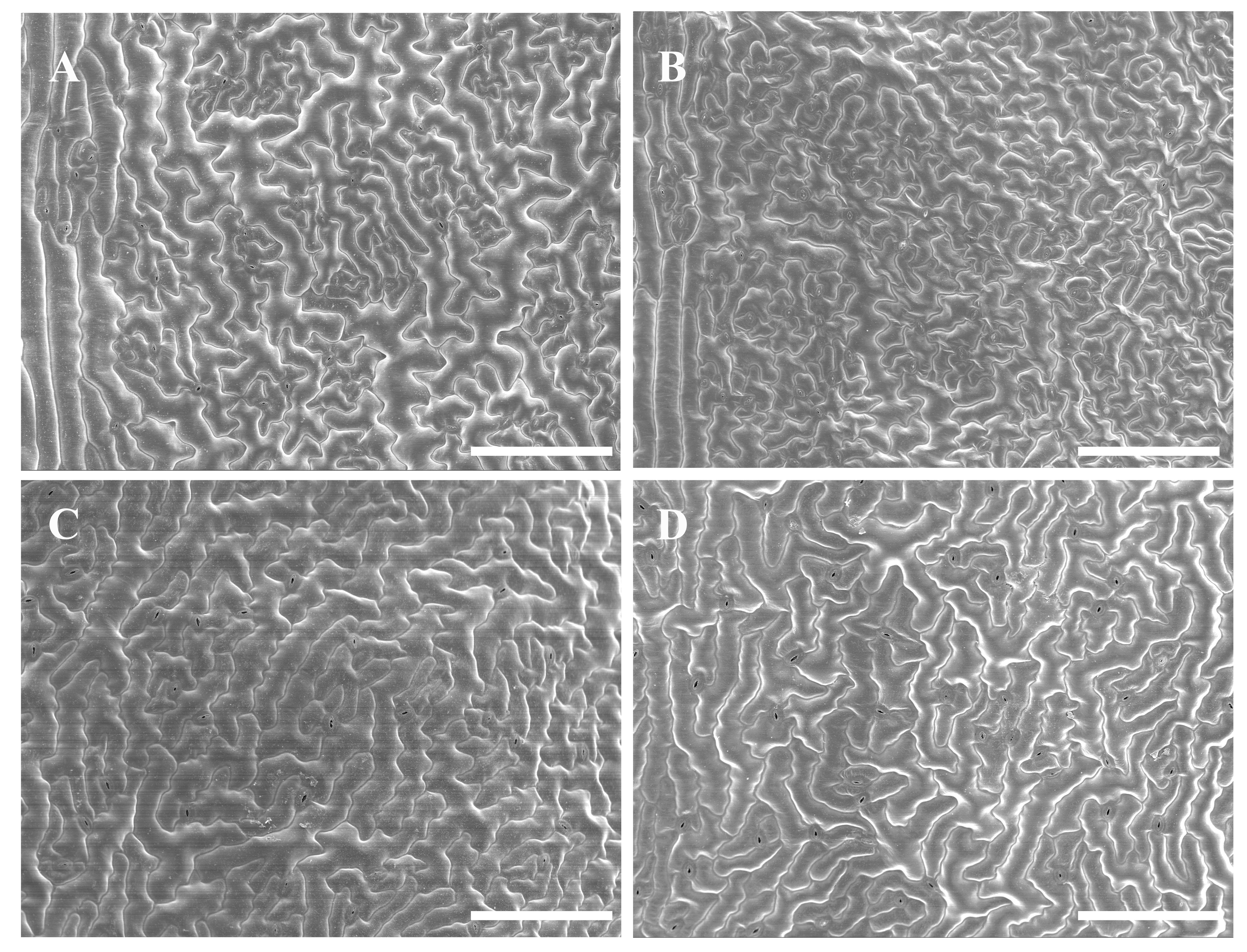

Supplement: Figure S5 — Loss of three functional copies of RBR in tetraploids does not lead to aberrant cell division and cell size in leaves. Shown are scanning electron micrographs of abaxial region of mature cauline leaves in (A) diploid Col wild-type (RBR/RBR), (B) diploid rbr mutant (RBR/rbr), (C) tetraploid Col wild-type (RBR/RBR/RBR/RBR) and (D) rbr triplex (RBR/rbr/rbr/rbr). Scale = 90 µm. (6.86 MB TIF) [file pgen.1000988.s005.tif]
