# Supplementary material for: Dosage-Sensitive Function of RETINOBLASTOMA RELATED and Convergent Epigenetic Control Are Required during the Arabidopsis Life Cycle
Source: PLoS Genet. 2010 Jun 17;6(6):e1000988. doi: 10.1371/journal.pgen.1000988 (PMC2887464; doi:10.1371/journal.pgen.1000988)
Supplement: Figure S2 — Schemes of tetraploid genetics dissecting RBR function. (A) RBR mediated triploid bridge led to autonomous tetraploidization of diploid plants heterozygous for rbr. Shown are representative flow cytometry histograms depicting the cellular ploidy of young cauline leaves. (B) Progeny testing (n = 103) of a tetraploid rbr heterozygote identified rbr triplex plants (shaded in yellow) (see Table 1 for details). (C) A second generation progeny test of an rbr triplex plant (n = 93) (see Figure S4 for additional data). (2.17 MB DOC) [file pgen.1000988.s002.doc]

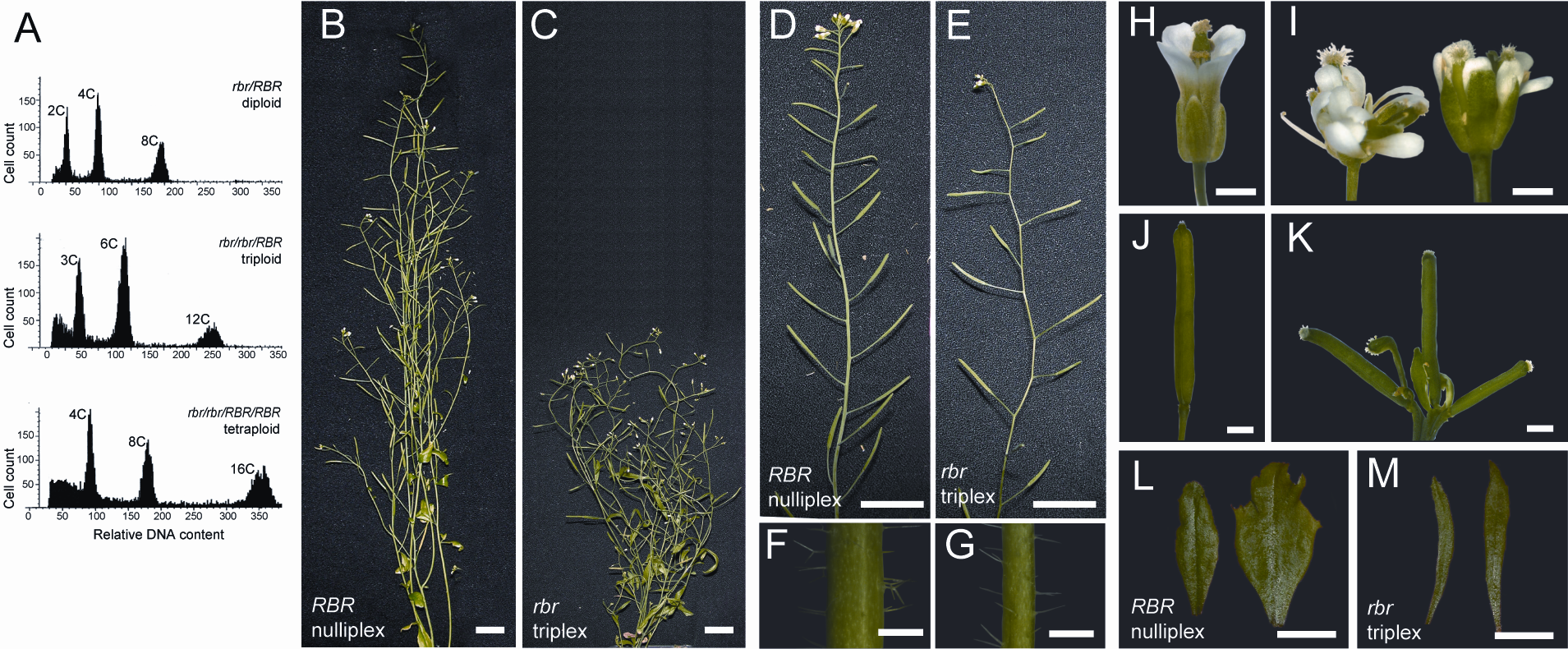


*rbr/rbr/RBR/RBR*

*(1 plant)*

*rbr/RBR/RBR/RBR*

*41 plants*

*rbr/rbr/RBR/RBR*

*52 plants*

*rbr/rbr/rbr/RBR*

*2 plants*

*RBR/RBR/RBR/RBR*

*8 plants*

*rbr/rbr/rbr /RBR*

*1 plant*

**C**

*rbr/RBR/RBR/RBR*

*14 plants*

*rbr/rbr/RBR/RBR*

*74 plants*

*rbr/rbr/RBR/RBR*

*5 plants*

**A**

**B**
