# Supplementary material for: Dosage-Sensitive Function of RETINOBLASTOMA RELATED and Convergent Epigenetic Control Are Required during the Arabidopsis Life Cycle
Source: PLoS Genet. 2010 Jun 17;6(6):e1000988. doi: 10.1371/journal.pgen.1000988 (PMC2887464; doi:10.1371/journal.pgen.1000988)
Supplement: Figure S6 — Quantitative reduction of RBR in tetraploids does not lead to changes in ploidy of leaf and trichome cells. (A) Leaf ploidy in tetraploid wild type (RBR/RBR/RBR/RBR) and rbr triplex (rbr/rbr/rbr/RBR) recorded by flow cytometry (B) Ploidy classes of the trichomes in reference to the tetraploid (4C) guard cells, upon nuclear DNA quantification by confocal microscopy. (0.51 MB PPT) [file pgen.1000988.s006.ppt]

## Slide 1
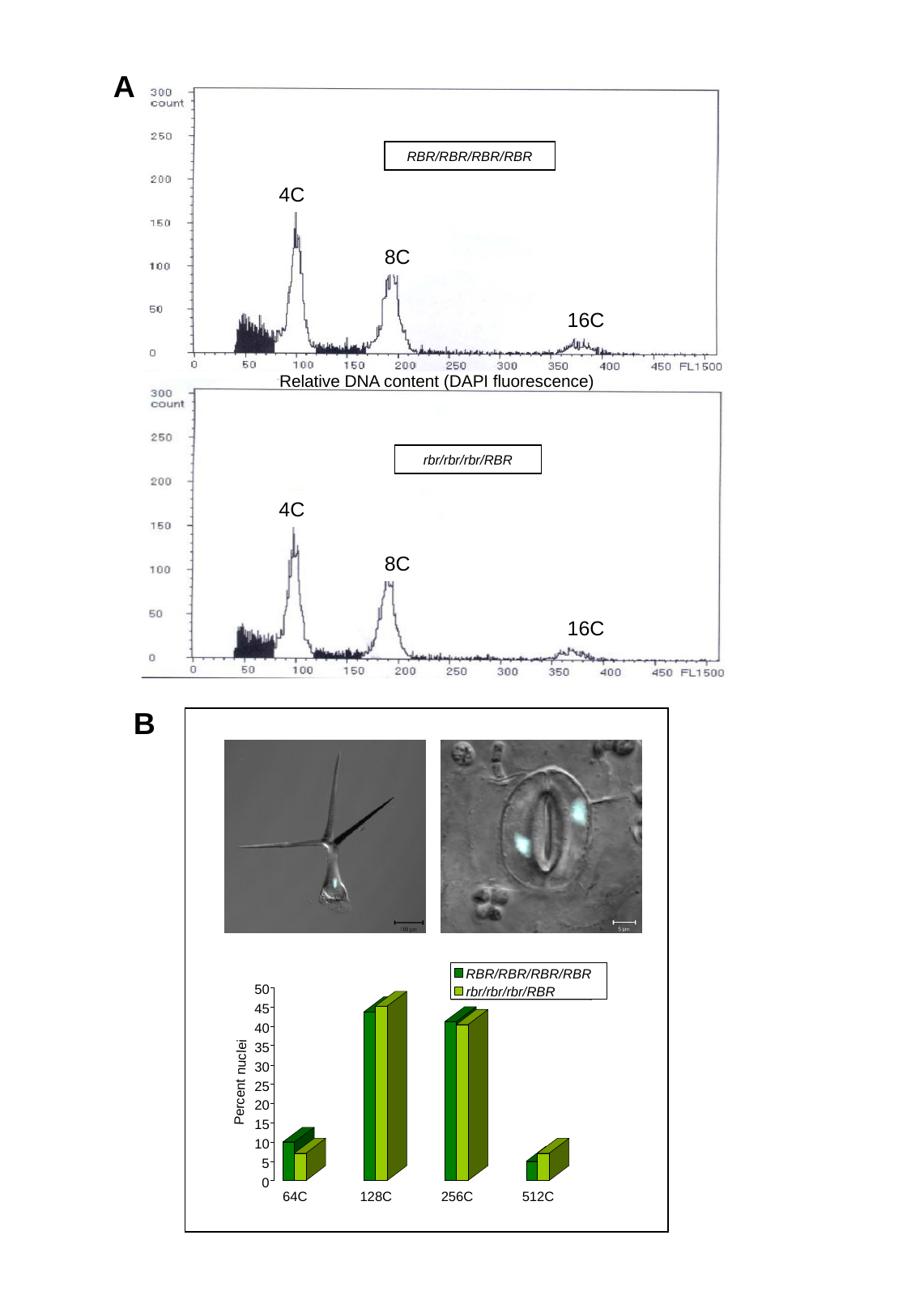

A
RBR/RBR/RBR/RBR
4C
8C
16C
Relative DNA content (DAPI fluorescence)
rbr/rbr/rbr/RBR
4C
8C
16C
B
RBR/RBR/RBR/RBR
50
rbr/rbr/rbr/RBR
45
40
35
30
Percent nuclei
25
20
15
10
5
0
64C
128C
256C
512C
