# Supplementary material for: Dosage-Sensitive Function of RETINOBLASTOMA RELATED and Convergent Epigenetic Control Are Required during the Arabidopsis Life Cycle
Source: PLoS Genet. 2010 Jun 17;6(6):e1000988. doi: 10.1371/journal.pgen.1000988 (PMC2887464; doi:10.1371/journal.pgen.1000988)
Supplement: Table S1 — Primers used in quantitative real time PCR assays. (0.03 MB DOC) [file pgen.1000988.s007.doc]

**Table S1.** Primers used in quantitative real time PCR assays

| **Primer sequence [F – forward; R – reverse]** | **Purpose** |
| --- | --- |
| F: CCA TCA TAC TCA TTG CTG ATC C  R: TCC ATC AGG TCA ACA GCT TG | *rbr-3* genotyping, T-DNA left-border |
| F: CCC AAA TAG TTC CCT TGG TG  R: TCA TCC ATC AGG TCA ACA GC | *rbr-3* genotyping, WT genomic spanning the T-DNA insertion site |
| F: TCC TCT TCG GGG ATC AAA GAT G  R: AGG CTA TTT GCT ACC ATG GAA TCG | *RBR*; RT-PCR |
| F: GAG ATA AAC TGA AAT TCG CCA ACC  R: CTC GAT CTG GCT CAT ACC GGT | *CLF*; RT-PCR |
| F: CCC AAT TGC TAC GCT AAG GT  R: GCT CTT CGC TAG CTT CTA TTC G | *SWN*; RT-PCR |
| F: TTG AGC CCT TCT CTC TCT GC  R: GGG TGA ATC CAA CGG TAA AA | *VRN2*; RT-PCR |
| F: AGA TTG CAC GGA CCC ATC AT  R: GTT CCA CAG TTT CAC CAT AAA CA | *EMF2*; RT-PCR |
