# Supplementary material for: Dosage-Sensitive Function of RETINOBLASTOMA RELATED and Convergent Epigenetic Control Are Required during the Arabidopsis Life Cycle
Source: PLoS Genet. 2010 Jun 17;6(6):e1000988. doi: 10.1371/journal.pgen.1000988 (PMC2887464; doi:10.1371/journal.pgen.1000988)
Supplement: Text S1 — Tetraploid genetics and double reduction. (0.09 MB DOC) [file pgen.1000988.s008.doc]

**Supplementary Text**

**Tetraploid genetics and double reduction**

In meiosis of diploid organisms, homologous chromosomes form bivalents, where recombination happens; this event is followed by two rounds of cell divisions giving rise to the gametes. Meiosis I is a reductional division when sister chromatids of the same chromosome go to same pole, therefore reducing the chromosome number to the half; and subsequent meiosis II is an equational, mitotis-like division when sister chromatids separate and go to the opposite poles.

In tetraploid meiosis, the four homologous chromosomes can form a quadrivalent. If chromosomes in quadrivalent have recombination between centromere and the investigated locus, the two pairs of chromatids resulting from such crossing-over can pass to the same pole in anaphase I, creating possibility for the two sister alleles to be included in the same gamete. This situation is called double reduction (Figure A).


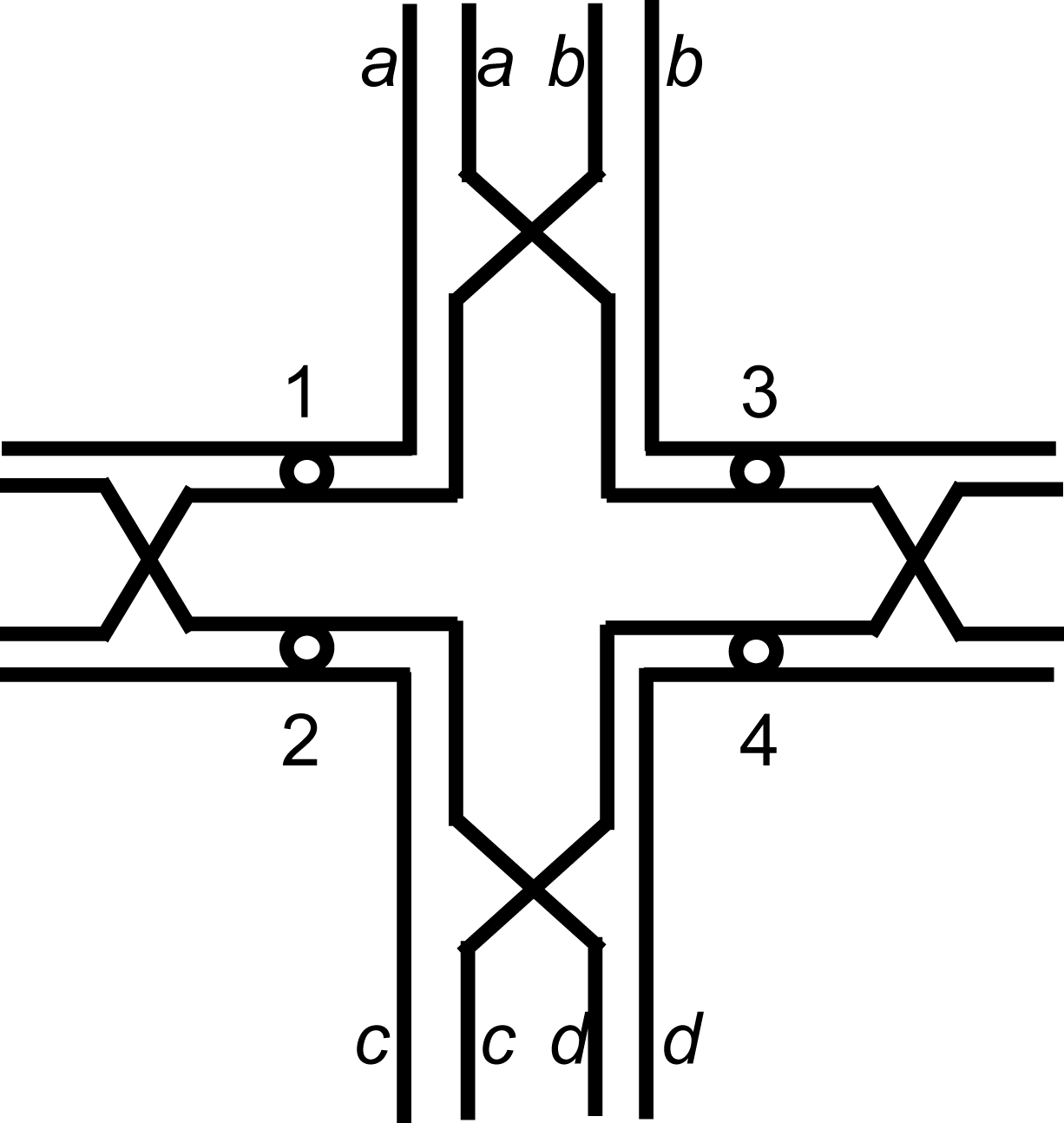


**Figure A.** Recombination in quadriplex. Centromeres of the four chromosomes marked as circles 1-4; the locus of interest as *a,b,c,d* [1].

There are three possible different arrangements (n-1, where n is chromosome number in quadrivalents) of the four alleles *a, b, c* and *d* (Table A).

**Table A.** An example of quadrivalent allele segregation and double reduction in gametes.

|  | Segregation types | | |
| --- | --- | --- | --- |
|  | alternate | adjacent | adjacent |
| At end of meiosis I |  |  |  |
| centromere disjunctions | 1+4  2+3 | 1+3  2+4 | 1+2  3+4 |
| chromatid pairs at the poles | *ab, cd*  *cd, ab* | *ab, ab*  *cd, cd* | *ab,cd*  *cd,ab* |
| At end of meiosis II |  |  |  |
| gametes | 2(*ac+bd+ad+bc)* | *aa+bb+2ab+cc+dd+2cd** | 2(*ac+bd+ad+bc)* |

* note that the *aa, bb, cc* and *dd* double reductional combinations are results of one of adjacent segregations.

Considering the two other possible arrangement of the alleles, the total gametic genotypes are: 10 *ab +* 10 *ac +* 10 *ad* *+* 10 *bc* + 10 *bd* + 10 *cd* + 3 *aa* + 3 *bb* + 3 *cc* + 3 *dd*. The last four combinations with sister chromatids comprise 12 out of total 72 combinations; α =12/72 or 1/6 is the maximum value of double reduction [1].

The four double reduction combinations *aa, bb, cc* and *dd* occur with frequency of α/4 each; the total frequency of the six other combinations *ab,* *ac, ad*, *bc, bd* and *cd* is 1-α and therefore (1- α)/6 for each of them.

As an example, in our study, we calculated gametic genotypes of *rbr* triplex *rbr/rbr/rbr/RBR* corresponding to the alleles *a/b/c/d* (Table B).

***Table B.*** *Gametic frequency in rbr triplex rbr/rbr/rbr/RBR [1]*.

| Double reduced gametes with two alleles from the same chromosome | | | Gametes with two alleles  from different chromosomes | | |
| --- | --- | --- | --- | --- | --- |
| Combinations  *a/b/c/d* | *rbr/rbr/rbr/RBR* | Frequency | Combinations  *a/b/c/d* | *rbr/rbr/rbr/RBR* | Frequency |
| *aa* | *rbr/rbr* | α/4 | *ab* | *rbr/rbr* | (1- α)/6 |
| *bb* | *rbr/rbr* | α/4 | *ac* | *rbr/rbr* | (1- α)/6 |
| *cc* | *rbr/rbr* | α/4 | *ad* | *rbr/RBR* | (1- α)/6 |
| *dd* | *RBR/RBR* | α/4 | *bc* | *rbr/rbr* | (1- α)/6 |
|  |  |  | *bd* | *rbr/RBR* | (1- α)/6 |
|  |  |  | *cd* | *rbr/RBR* | (1- α)/6 |
|  | total | α |  | total | 1- α |

By summing up all the combinations, the frequencies of the three types of gametes in the triplex are reduced to

(2+α)/4 *rbr/rbr* + (2-2α)/4 *rbr/RBR* + α/4 *RBR/RBR*

The corresponding frequencies of gametes for *rbr* duplex and simplex were calculated the same way. Ratios for different progeny genotypes were calculated using Punnett square.

**Reference:**

1. Burnham CR (1964) Discussions in Cytogenetics. Minneapolis, USA: Burgess Publishing Company. 375 p.
