# Supplementary material for: Dosage-Sensitive Function of RETINOBLASTOMA RELATED and Convergent Epigenetic Control Are Required during the Arabidopsis Life Cycle
Source: PLoS Genet. 2010 Jun 17;6(6):e1000988. doi: 10.1371/journal.pgen.1000988 (PMC2887464; doi:10.1371/journal.pgen.1000988)
Supplement: Supplementary file 6 [file pgen.1000988.s004.doc]

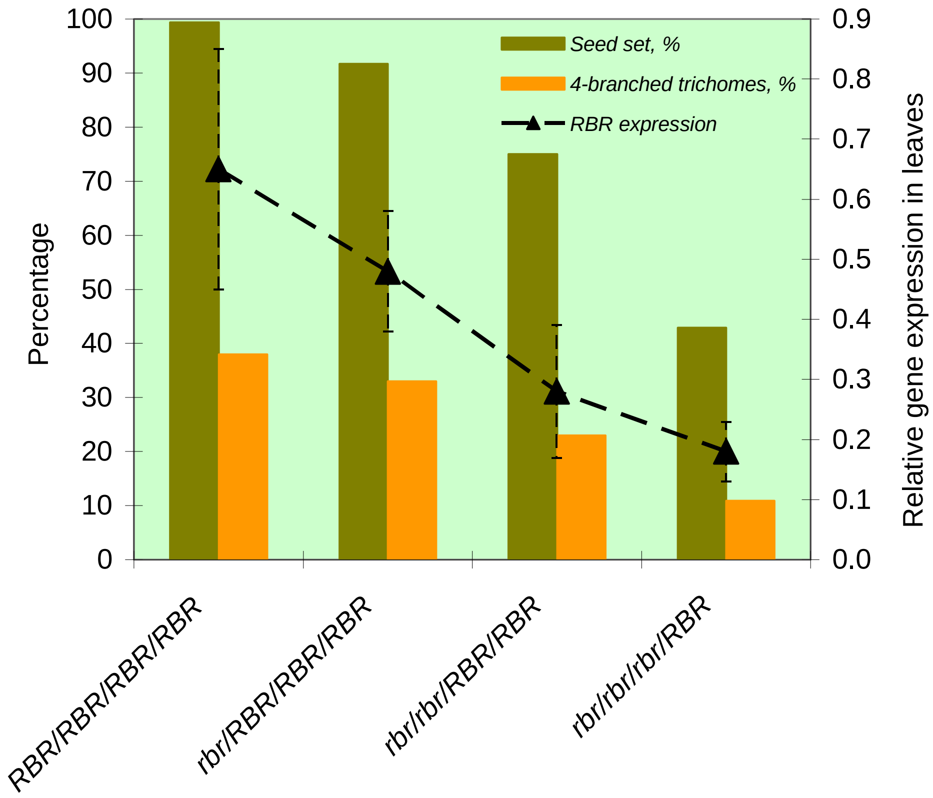

Seed set, %
4-branched trichomes, %
RBR expression
Percentage
Relative gene expression in leaves
100
90
80
70
60
50
40
30
20
10
0
0.9
0.8
0.7
0.6
0.5
0.4
0.3
0.2
0.1
0.0
RBR/RBR/RBR/RBR
rbr/RBR/RBR/RBR
rbr/rbr/RBR/RBR
rbr/rbr/rbr/RBR
